# Dietary changes based on food purchase patterns following a type 2 diabetes diagnosis

**DOI:** 10.1017/S1368980022001409

**Published:** 2022-10

**Authors:** Anna Kristina Edenbrandt, Bettina Ewers, Heidi Storgaard, Sinne Smed

**Affiliations:** 1 Department of Economics, Swedish University of Agricultural Sciences, Uppsala 750 07 Sweden; 2 University of Copenhagen, Department of Food and Resource Economics, Copenhagen, Denmark; 3 Steno Diabetes Center Copenhagen, Copenhagen, Denmark

**Keywords:** Event study analysis, Behavioural change, Dietary change, Purchase patterns, Type 2 diabetes

## Abstract

**Objective:**

The study explores whether type 2 diabetes (T2D) diagnosis affects food consumption patterns in line with the dietary recommendations provided to individuals in relation to a diagnosis.

**Design:**

Based on detailed food purchase data, we explore which dietary changes are most common following a T2D diagnosis. Changes are investigated for several energy-adjusted nutrients and food groups and overall adherence to dietary guidelines.

**Setting:**

We use data on diagnosis of T2D and hospitalisation in relation to T2D for a sample of adult Danes registered in the official patient register. This is combined with detailed scanner data on food purchases, which are used as a proxy for dietary intake.

**Participants:**

We included 274 individuals in Denmark who are diagnosed during their participation in a consumer panel where they report their food purchases and 16 395 individuals who are not diagnosed.

**Results:**

Results suggest some changes in dietary composition following diagnosis, as measured by a Healthy Eating Index and for specific food groups and nutrients, although the long-term effects are limited. Socio-economic characteristics are poor predictors of dietary changes following diagnosis. Change in diet following diagnosis vary with the pre-diagnosis consumption patterns, where individuals with relatively unhealthy overall diets prior to diagnosis improve overall healthiness more compared to individuals with relatively healthy diets prior to diagnosis.

**Conclusions:**

Adherence to dietary advice is low, on average, but there is large variation in behavioural change between the diagnosed individuals. Our results stress the difficulty for diagnosed individuals to shift dietary habits, particularly in the long term.

Due to unhealthy eating, sedentary lifestyles and ageing populations, the proportion of individuals with lifestyle-related diseases and obesity is increasing^(1,2)^. In 2016, more than 1·9 billion adults where overweight. Of these, 650 million were obese^(3)^. Type 2 diabetes (T2D) is a potential consequence of poor diet and obesity^(4)^, since obesity and inactivity cause accumulation of visceral fat leading to metabolic changes, which results in insulin resistance and over time impaired in insulin secretion, resulting in impaired glucose tolerance, prediabetes and eventually T2D^(5)^. With T2D, the risk of severe and life-threatening microvascular complications (retinopathy, nephropathy and neuropathy) and macrovascular complications (CVD) markedly increases^(6)^.

Globally, approximately 463 million individuals are diagnosed with diabetes, and it is the direct cause of 4·2 million deaths per year^(2)^. This induces a huge burden on the health care system, and the total costs related to diabetes constitutes 10 % of global health expenditures, with the major part being treatment of complications^(2,7)^. In Europe alone, 60 million individuals are diagnosed with diabetes, and the prevalence is increasing in all age groups^(4)^. It is estimated that in Denmark, the prevalence of individuals at increased risk of diabetes (prediabetes) is approximately 360·000^(8)^, and that at least 250·000 individuals have overt T2D^(9)^ and the costs related to T2D is estimated to 87 million DKK a day^(10)^. Both prevalence and extent of obesity and thereby diabetes continuously increase worldwide, and projections point to catastrophic consequences for health care systems and societies^(2,3)^. There is thus a need of increased focus on prevention and treatment^(10)^.

According to both international^(11)^ and Danish treatment guidelines^(12)^, the key components in both prevention and treatment of T2D are lifestyle modifications and pharmacological treatment. The impact of adhering to the established food-based dietary guidelines on morbidity and mortality in the general population has been studied extensively in several primarily prospective cohorts^(13–19)^. All pointing at a reduced risk of T2D, CVD and all-cause mortality among individuals with higher adherence to the established dietary guidelines. Similarly, there is a reduced risk of CVD and mortality following a T2D diagnosis due to lifestyle modifications^(19–23)^. Thus, eating healthily is essential, especially following a T2D diagnosis. Accordingly, healthy dietary habits focusing on carbohydrate quality and quantity (e.g. high in dietary fibre and with limited added sugar) and fat quality (with limited intake of saturated fat and high intake of monounsaturated fat) are recommended in both national and international guidelines for the management of diabetes^(24–26)^.

Despite dietary guidelines for the management of T2D based on evidence of the effects on glycaemic and metabolic control, and reductions in diabetes-related complications, studies show that adherence to the dietary guidelines is rather poor among individuals with diabetes^(27–30)^. However, prevalent research examining dietary intake among individuals diagnosed with T2D is commonly based on proxies for diagnosis status such as self-tests^(31,32)^, self-reported diagnosis status^(33–35)^ and/or self-reported dietary intake^(34–37)^. Yet, self-reported data are associated with uncertainty, and underreporting is a major problem in self-reported dietary assessment methods, especially among obese including individuals with T2D^(27,38,39)^. Furthermore, much of the analysis is based on cross-sectional or repeated cross-sectional analyses, comparing diagnosed individuals with undiagnosed individuals. An exception is the seminal study on dietary changes following T2D by Oster^(32)^, who found a small average reduction in energy intake, but with substantial variation between individuals based on a panel dataset on observed food purchases. Oster uses the purchase of diabetes-related products (products such as testing strips and glucose monitors) and a machine learning approach to identify diabetes diagnosis.

There are three main contributions of this paper. First, we extend on the work by Oster^(32)^ by utilising an identification strategy for the T2D diagnosis of significantly higher precision. We use data on diagnosis for a sample of Danish adults registered in the official patient register. Second, in contrast to many of the existing studies, we use detailed observed data on food purchases as a proxy for dietary intake. The detailed purchase data also allow us to investigate which dietary changes are most common among individuals following a T2D diagnosis. Changes are investigated for several energy-adjusted nutrients and food groups as well as overall adherence to dietary guidelines. Importantly, the panel structure of our data enables us to explore if the potential changes in purchase patterns are of short- or long-term duration. Most of the existing literature are based on cross-sectional observations or shorter timer periods. Third, we explore differences between sociodemographic profiles in relation to changes in food consumption patterns following a T2D diagnosis.

## Materials

We combine several sources of data, which enables us to explore dietary effects from T2D diagnosis. The data include detailed information on food purchases of the households in a large consumer panel. Further, we include information about the point in time for T2D diagnosis for individuals in each household in the consumer panel. Finally, the data include information about the socio-economic profile of the individuals within the households.

## Food purchase data

The analysis is based on a dataset provided by GfK ConsumerScan Denmark that covers the period 2006–2017. The dataset consists of approximately 1500 households per week that register all food products purchased on a daily basis with a home scanner device, providing information on product level. Each month some households leave the panel and others enter. Gfk seeks to hold a representative sample of households with respect to age, education, family size and geographical location, so leaving households are replaced by households with similar characteristics. A total of 8524 households and 16 395 adult individuals participated in the panel during all or some of or the period included in this study.

The purchase data are combined with nutrient content data based on the Danish Food Composition Databank managed and updated by the National Food Institute^(40)^. This databank includes information about the content of energy and macronutrients (e.g. carbohydrates, protein and fat) as well as subcategories of macronutrients (e.g. sugar, fibre, saturated, monounsaturated and polyunsaturated fat) content per 100 g of each of the 1049 food products included in the databank at the point of establishment of the dataset. The database is continuously updated to include more foods^(40)^. For the type of foods that do not exist in the databank, the nutritional contents are calculated based on an average recipe for the food in question.

Since we only have information about food purchases for the entire household, we construct individual consumption for each member of the household based on standard individuals. Each household member is given a weight relative to the standard individual, dependent on gender and age. The recommended daily energy intake is taken from the Danish National Survey of Diet and Physical Activity in Denmark and Becker with colleagues^(41,42)^. The survey consists of energy intake for sedentary and active females and males in various age groups. The standard person chosen here is a woman at the age of 30–60 years with average exercise levels. She has a recommended energy requirement at 9900 kJ/d. That is, a household consisting of a female and a male both aged 30–60 years will have a family energy requirement of 11 000 kJ + 9900 kJ = 20 900. This household hence consists of 2·1 standard persons. As a robustness test, we also estimate models based on a sample consisting of only single households.

We employ two types of outcome measures for dietary healthiness. The first type of measure is a composite measure in the form of a Healthy Eating Index (HEI), evaluating the adherence to the official dietary guidelines of the Danish Ministry of Family and Consumer Affairs, taking the composition of the household’s diet into account. The HEI, developed by Smed^(43)^ is similar in set-up as the HEI originally developed by Kennedy et al.^(44)^ but adjusted to the Danish dietary recommendations, gives an indication of the dietary healthiness of the household covering eight aspects of the diet, including the amount of fruit and vegetables, fish and the sugar, fat and fibre content. The original HEI ranges from 0 to 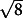 but is rescaled to range from 1 to 100 for ease of interpretation. More details on the criteria that are included in the construction of the HEI are available in Appendix A.1.

The second category of dietary outcome measure is a set of variables based on energy percentages from specific nutrients or product types. The analysed nutrients were selected based on the recommendations to individuals with diabetes in Denmark and include the energy percentages from fruit and vegetables, fish, meat and three different unhealthy food products (sugar-sweetened beverages (SSB), cakes and candy). Moreover, we use the energy percentages from protein, unsaturated fat, saturated fat, added sugar, carbohydrates and fibre. These variables are calculated as grams of the nutrient consumed times the energy density in the nutrient in kJ/g and then divided by total energy consumption in the household. Each of the dietary measures is calculated on a monthly basis.

In addition, as a robustness test, we estimate models for the specific food and nutrient categories, where the unit is the absolute intake of energy rather than the percentage of energy. The energy from each category was adjusted to follow standard individual units for each individual.

## Diagnosis data and identification

The identification of T2D diagnosis accommodates individuals who have been diagnosed at a hospital and/or had a complication related to T2D, treated at a hospital. The national patient register provides these data. The time of the diagnosis is registered in the data. For individuals who do not have a diagnosis registered, we use the first occurrence of a T2D-related complication as identification of diagnosis. This is made possible from the detailed data on treatments at hospitals. For example, when an individual is treated for a foot or eye complication, the medical doctor at the hospital registers it as ‘foot/eye complication in relation to T2D’. These data cover the period 1990–2017. Moreover, we obtain information about prescription of medication for T2D treatment provided by the Danish Medicines Agency. For individuals treated with T2D medicine, but without a diagnosis or complication registered in our data, we use first occurrence of prescribed T2D medicine as identification of a diagnosis. The medicine that is used as identifier is insulin (all types). This includes all medicines coded as A10 in the medicine register^(45)^. Individuals who are diagnosed with type 1 diabetes (T1D) are not included in this study. We test the robustness of this identification strategy by also estimating the model with a more strict definition of T2D diagnosis, where those who are only medicated are excluded from analysis. Diagnosis made by general practitioners (GP) are not registered in our data. However, the GPs’ medical prescription is included. For the purpose of identification, it is important to note that individuals who are diagnosed by a GP, and neither are treated for any complication nor prescribed any medicine, are not identified as individuals with T2D in our sample. This implies that if such individuals change behaviour in a systematically different way compared to those identified in our sample, this would cause bias in our estimations. We expect that individuals who are not treated with T2D medicine or for complications are more successful in adapting their lifestyle, including their diet, in accordance with the recommendations. This implies a downward bias in the estimates of behavioural change in our data.

In Denmark, lifestyle intervention programmes for individuals are offered by both the health care system and the municipalities in conjunction a with T2D diagnosis. These include education regarding the disease and self-management, diet, physical activity/exercise training and smoking cessation^(12)^. Thus, a T2D diagnosis in Denmark is associated with the information treatment from the diagnosis itself, where the diagnosed individual is made aware of the medical status. Further, a T2D diagnosis also implies that the individual is offered more detailed information from dietarians on practical guidelines to target the disease. This study explores the effects from the information treatment from the diagnosis itself, while we do not have information about if the individual in addition participate in follow-up sessions with dietarians.

## Coupling of food purchase data and register data

The food purchase data were connected with the official register data by Statistics Denmark based on a search for CPR numbers (personal identity number from the Central Person Register) of the individuals in the household panel. GfK provided names, addresses and ages of the panel members, and Statistics Denmark used this information to retrieve the CPR numbers and thereafter to match with register data. All CPR numbers, names and addresses were replaced with a unique identification number, ensuring that the households are anonymous in the dataset that we access. Data are only available through the server at Statistics Denmark, and only estimation results and average statistics can be exported from the server.

Our final dataset includes 1296 individuals who have T2D (about 8 % of the individuals in the panel), which is comparable to the rate of T2D in the Danish population (about 5 % of total population), when we take into account that our panel members are older than the average Danish population. In the main analysis, we only include individuals who are diagnosed during their participation in the consumer panel, such that they are included if they register purchases at least one of the 3 months prior to the diagnosis, and at least one of the 3 months following their diagnosis. This gives us 274 individuals who were diagnosed with T2D. Individuals diagnosed prior to their participation in the food panel are excluded from the main analysis.

## Registry data on personal information

To explore heterogeneity in dietary changes upon a diagnosis, we link the purchase data and diagnosis data with registry data from Statistics Denmark on personal characteristics such as age, gender, family composition and income.

According to the literature concerning information provision in the general population, women are more prone to use and respond to health labels and claims, and they are generally more interested in healthy eating and information about healthy eating^(46,47)^. Hence, we expect that women have a larger response to the information provided in relation to a T2D diagnosis compared to males.

In general, income and higher education are associated with healthier diets^(48)^, and these groups are also found to be more responsive to information and to a larger degree use and understand labels^(46)^. We therefore expect a positive relationship between the reaction to a diagnosis and income.

We also include age, although there is not clear evidence to suggest any particular effect of age on the response to information^(46)^ and therefore not on the reaction to a diagnosis. Further, we explore whether the reaction to a T2D diagnosis varies between individuals in single and multiple individual households. Food consumption is an important part of an individual’s social life. A diagnosed individual may make changes in diet, but if the rest of the household does not make such changes, the dietary changes measured on the household basis are less clear.

In sum, since we only have weak prior expectations that are based on the general publics’ reaction to information, we choose an exploratory approach, as we do not have clear evidence to guide expectations regarding the influence of socio-economic characteristic on the response to the information given in relation to a diagnosis.

Summary statistics for the sample are presented in Table 1. The sample of individuals who are diagnosed with T2D during their participation in the panel are compared with individuals without a known T2D diagnosis. Individuals with T2D are significantly older with a mean age of 60 years compared with 45 years for individuals without known diabetes. Moreover, individuals with T2D consists of smaller households, which presumably is related to age, where fewer have children living in the household. We do not see statistically significant differences in gender distribution or income levels between individuals with and without known diabetes.

**Table 1:** Summary statistics

	T2D	No known T2D	Difference
Individuals	274	15 099	
Personal characteristics			
Female (%)	55·1	53·7	ins
Age (mean)	59·9	44·8	***
Household size (pers)	1·9	2·5	***
Single household (%)	34·3	17·9	***
Annual personal income (DKK)	257 645	262 934	ins
Food purchase patterns			
HEI (running from 0 to 100)	75·7	76·3	ins
Food categories, percent of total energy consumed			
Fruit and vegetables	6·4	8·2	*
Fish	1·3	1·1	ins
Meat	13·5	10·8	***
Sugar-sweetened beverages	2·4	2·5	ins
Cakes	2·5	2·4	ins
Candy	2·2	3·1	*
Nutrients, percent of total energy consumed			
Protein	14·9	14·8	ins
Unsaturated fat	19·3	18·1	ins
Saturated fat	16·3	14·9	***
Added sugar	4·5	5·4	**
Carbohydrates	40·7	44·5	***
Fibre	2·1	2·2	ins

T2D, type 2 diabetes; HEI, Healthy Eating Index.

Food purchase patterns for the T2D sample are calculated based on the pre-diabetes period only.

Tests for differences between samples (*t* tests and 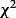 tests):

* indicates if *P* < 0·05,

** if *P* < 0.01, and

*** if *P* < 0.001, ins = insignificant. (10 DKK∼1.6 USD).

We further compare the purchase patterns between individuals with T2D and without known T2D (bottom panel in Table 1). The purchase patterns are based on household basis. This implies that non-T2D-individuals who are in a household with a T2D individual are included in the non-T2D sample, when they have the same purchase patterns as their T2D household members. However, these individuals constitute a minor share of the total number of individual in the non-T2D sample. Only the months of purchases prior to the diagnosis for individuals with T2D are included to exclude any effect on purchases from information provided when diagnosed. Surprisingly, there is not a statistically significant difference in the overall dietary healthiness, as measured by the HEI. However, some purchase patterns vary significantly between those with and without known T2D. Individuals with T2D purchase less fruit and vegetables and more meat. Their purchases also contain a higher share of saturated fat, while less carbohydrates and added sugar.

## Methods

### Empirical framework

To explore the dietary changes in relation to a diagnosis, we apply an event study analysis using the panel structure of the data. This allows us to examine whether T2D individuals change their food purchases in accordance with the dietary recommendations provided when diagnosed (hereafter referred to as ‘information treatment’). The analysis only includes individuals who were diagnosed (following the identification strategy in section 3·2) during their participation in the food panel and individuals with no diagnosis as a control group. Individuals who are diagnosed prior to joining the panel are excluded from analysis. We test if the assumption of parallel trends pre-diagnosis hold.

We test for information treatment effects on a number of diet-related measures, as described in section 3·1. For this reason, equation (1) describes a general model, where the dependent variable *y* represents the different measures investigated. We estimate the following specification:(1)
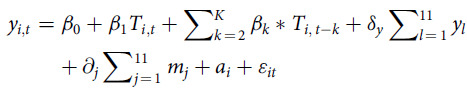




Let 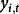 equal the dietary measure for individual i during month t. We include yearly *(* 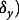 and monthly 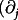 ) fixed effects, to control for shifts in diet over the time span of the data, as well seasonal effects. Several new dietary ‘trends’, for example, the New Nordic Diet, are observed during our data period. These might lead to systematic changes in dietary composition, which is captured through the yearly dummies. Monthly dummies are introduced in order to capture seasonality in healthiness of diets^(49,50)^. The term *a*_*i*_ is an individual fixed effect and 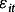 are regression disturbances with mean zero that are independently distributed. To account for general forms of autocorrelation in 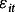 across months for an individual, and the possibility that this pattern differs across individuals, we report test statistics based on standard errors that are robust to this form of heteroscedasticity and autocorrelation.

The variable of main interest is the information treatment effects variables (*β*). The information treatment is the diabetes diagnosis. The treatment variable is constructed as a dummy variable, taking the value one from the month an individual was diagnosed and onwards, and zero otherwise. To test for delayed or reverse effects following diagnosis, we include lagged variables such as 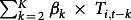 . Hence, the lagged treatment variable takes the value 1 if the individual was diagnosed *k* months prior. We initially include K = 12 lagged variables, since long-term follow-up of dietary changes for more than 12 months is important in trials examining the dietary impact on clinical outcomes. Significant improvements in, for example, glycaemic control among individuals with T2D have been difficult to find after 12 months, possibly due to lack of long-term dietary adherence^(51)^. We check robustness of our results by artificially setting the treatment date to be 1, 2, 3 and 6 months before the diagnosis. This is to test if diagnosed individuals are aware of their condition before the diagnosis, as identified with our identification strategy, and adjust their diet prior to their diagnosis in conjunction with a hospital visit.

In a second set of analysis, we test for heterogeneity in purchase behaviour following diabetes information treatment related to personal characteristics. For this purpose, we included the diagnosed individuals only and generate a variable for each diagnosed individual who described the individual’s diet before and after the diagnosis. For each of the dietary outcome variables (HEI, food and nutrient-specific energy shares), we calculate the average value over 6 months prior to the information treatment, as well as the average value over the 6 months after the information treatment. We generate the *individual change* variable ( 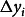 ) from the change in these averages for each individual:(2)
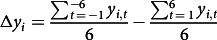

where *t* = 0 is the month of the diagnosis. For individuals with missing information for months prior or following the diagnosis, we calculate the average for the non-missing months. We do not include data based on the month of the diagnosis, since this month may not have been representative of the purchase behaviour. For example, individuals receiving their diagnosis during hospitalisation will likely do less grocery shopping during this month. We estimate the following specification to test for heterogeneity in the change in purchase patterns following a diagnosis:(3)
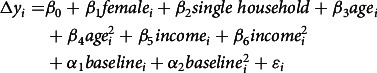




We estimate equation (3) by ordinary least squares. While the previous analysis based on equation (1) includes both diagnosed and non-diagnosed individuals, equation (3) includes diagnosed individuals only. We include the individuals purchase behaviour prior to diagnosis (*baseline* purchase behaviour), to investigate how the purchase behaviour prior to diagnosis relates to the behavioural change following the diagnosis. Hence, the terms denoted with 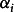 estimate the effect of the baseline purchase behaviour ( 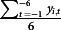 ). As robustness test, we also estimate equation (3) where the baseline consumption is divided into quartiles. We generate a *q25*-dummy variable that take the value 1 if the individual is among the lowest 25 % and 0 otherwise and correspondingly *q75*-dummy variable for the top 25 %. Furthermore, the results that we retrieve from estimating equation 3 could have been derived from estimating equation (1) with interactions between the treatment dummies and the sociodemographic variables. However, as we have a relatively small number of diagnosed individuals, this leads to many insignificant variables.

## Results

### Event study on effect of type 2 diabetes diagnosis

**Fig. 1:**
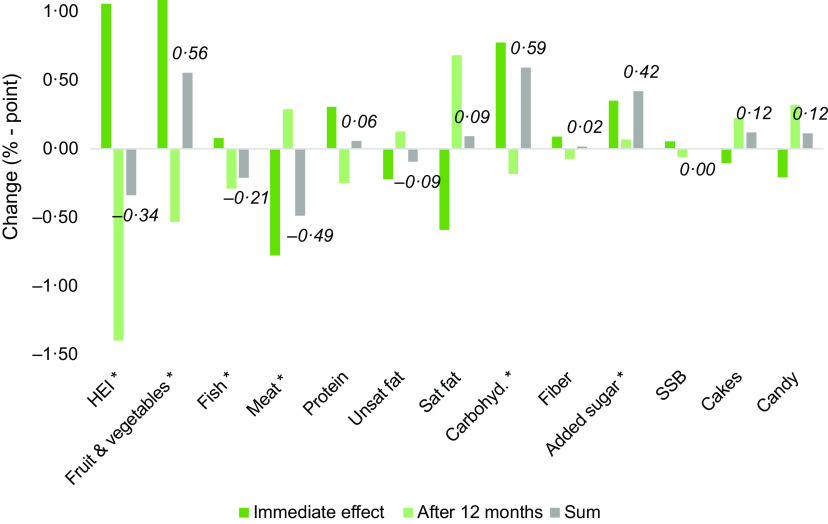
Effect of diagnosis on dietary health. *The sum of immediate and after 12 months changes is significant. Labels show aggregated effect after 12 months. SSB, sugar-sweetened beverages

Figure 1 reports the association between diagnosis and dietary measures, where each of the dietary measures are regressed on the T2D diagnosis (equation 1), while Table A2 in appendix reports full results. The main variables of interest are the immediate effect of the diagnosis (*Diagnosis*) and the longer-term effect (the 12-month lagged diagnosis variable *Diagnosis12m*). Initially, we included lags for up to 12 months following diagnosis (K = 12 in equation 1). However, sensitivity analysis suggested that only including one lag where k = 12 did not decrease model fit, wherefore we proceed with only including *Diagnosis12m*. To investigate if the effects on the dietary outcomes holds in the longer run, we test if the sum of the immediate effect and the lagged effect are different from zero. Test results are displayed as asterix (*) in Fig. 1 and in the last row of Table A2 in appendix. The overall results reveal a statistically significant effect from diagnosis on the overall dietary healthiness (HEI) in the months following diagnosis, although the change is a relatively small improvement (1 percentage point increase). However, the immediate improvement in HEI does not hold in the longer term, as revealed by the negative and significant lagged effect from the diagnosis. The results rather suggest a worse HEI-value after 12 months.

While there exists regional variations in the dietary recommendations delivered by dietitians in Denmark, a number of key elements are consistently provided to T2D-diagnosed individuals. Such key elements in dietary management of T2D include improvement of overall carbohydrate quality (and quantity) by increasing the intake of dietary fibre from wholegrain products, eating more vegetables and some fruits, and limiting the intake of added sugar. In addition, emphasis is made on improving fat quality by increasing the intake of primarily plant-based MUFA and limiting the intake of SFA^(24–26)^.

In line with such dietary guidelines, we observed that the share of energy originating from fruit and vegetables increases following a diagnosis. Although these changes are somewhat reversed after the first 12 months, there is still a significant long-term increase in fruit and vegetable consumption. Intake of fish and meat declines on a long-term basis following diagnosis. The effects on unhealthy food groups show that while intake of candy and snacks decline in the short run, these effects are reversed in the longer run. However, the joint effect is nonsignificant, so the intake of SSB, cakes and candy are unchanged following diagnosis in the long run.

The energy share deriving from saturated fat decreased following diagnosis, which is in line with the recommendations. However, these changes are reversed after 12 months, resulting in insignificant long-term effects. We find the same pattern for dietary fibre, with an immediate increase, which is not maintained in the longer term. No significant change is found for the energy share from unsaturated fat. In contrast, we find that the short- and long-term shares of energy consumption from added sugar and carbohydrates increased following a diagnosis, which is in contrast to the dietary guidelines. While the effects on specific unhealthy food categories do not display an increase, the increased intake of added sugar and carbohydrates can be related to increased intake of other food categories, perhaps food groups where the added sugar content is less salient.

### Heterogeneity in dietary outcomes following diagnosis

We proceed by analysing variation in dietary change following a diagnosis. *Change* variables are generated for each dietary outcome, which is the difference between the diet in the months following the diagnosis and the months prior to the diagnosis, as specified in equation (3). Summary statistics for these change variables show that on average, none of the dietary measures change (Appendix Table A3). However, each of these change variables have wide distributions, suggesting that there is large heterogeneity in the change in diet following a diagnosis. For example, the average individual increases the energy share from fruits and vegetables with 0·95 percentage points, while individuals in the high end (95th percentile) has increased it with 6·4 percentage points, and in the low end (5th percentile) *reduced* it with 4·9 percentage point.

To gain insights into this heterogeneity, we first display scatterplots with the diagnosis-induced change on the pre-diagnosis consumption level including simple linear and quadratic regression lines (Figure A1 in appendix). The plots confirm the large heterogeneity in the dietary change due to diagnosis, but also for most food and nutrient groups a negative relation in pre-diagnosis consumption level and some rather large outliers. We estimate equation (3) for each of the dietary outcome variables to investigate if there are systematic differences in the reaction to a T2D diagnosis, as explained by socio-economic characteristics and on the purchase behaviour prior to diagnosis (baseline consumption). Results for these estimations are displayed in Table 2 with baseline consumption in a quadratic specification (Panel A) and in quartiles (Panel B). We note that while the models with quadratic specifications have better model fit, these are more sensitive to outliers. For this reason, we present both specifications and focus on results that hold across both specifications.

**Table 2:** Change in diet 6 months after diagnosis compared to 6 months prior to diagnosis

		Energy shares from food groups					
	HEI	F&V	Fish	Meat	SSB	Cakes	Candy
Panel A: Continous specification							
Baseline	−1415*	−0·354*	−0·467***	−0·691***	−0·260***	−0·221**	−0·032
(|t-ratio|)	2120	2050	3950	5920	2550	3260	0·450
Baseline^2^	0·758	1800**	4291***	0·712***	−0·589	−2398***	−1948***
(|t-ratio|)	1700	3090	4040	2670	1050	8030	5790
R^2^	0·19	0·06	0·08	0·28	0·18	0·60	0·40
F-statistic^a^	7·23	2·11	2·87	12·33	6·78	46·62	21·32
Panel B: Categorical specification							
Baseline_q25	0·022***	0·004	0·001	0·038***	0·008*	0·008*	0·003
(|t-ratio|)	2·710	0·380	0·280	4·570	1·830	1·950	0·680
Baseline_q75	−0·030***	0·000	−0·003	−0·045***	−0·014***	−0·023***	−0·018***
(|t-ratio|)	−3·850	−0·020	−1·230	−5·380	−3·090	−4·950	−4·480
R^2^	0·14	0·01	0·03	0·23	0·07	0·14	0·10
F-statistic	6·62	0·43	1·25	12·93	3·23	6·82	4·76

HEI, Healthy Eating Index, F&V, fruit and vegetables; SSB, sugar-sweetened beverages.

* *P* < 0.05,

** *P* < 0.01,

*** *P* < 0.001.

Only variables of interest is displayed here. Full regression tables are shown in supplementary material Table S11 and S12. t-Ratios are based on robust standard errors. For F-statistic of the null hypothesis, all coefficients are equal to zero.

*n* = 261.

**Table 2:** (Continued)

	Energy share from nutrients					
	Protein	Unsat. fat	Sat. fat	Carbo.	Fiber	Added sugar
Panel A: Continuous specification						
Baseline	0·005	0·016***	0·007	0·018**	0·001*	0·003
(|t-ratio|)	1·160	3·180	1·580	1·990	1·660	0·640
Baseline^2^	−0·012***	−0·021***	−0·022***	−0·028***	−0·004***	−0·011***
(|t-ratio|)	−3·070	−4·020	−4·730	−3·070	−5·340	−2·450
R^2^	0·07	0·14	0·13	0·10	0·15	0·05
F-statistic^a^	3·06	6·86	6·35	4·76	7·52	2·09
Panel B: Categorical specification						
Baseline_q25	0·005	0·016***	0·007	0·018**	0·001*	0·003
(|t-ratio|)	1·160	3·180	1·580	1·990	1·660	0·640
Baseline_q75	−0·012***	−0·021***	−0·022***	−0·028***	−0·004***	−0·011***
(|t-ratio|)	−3·070	−4·020	−4·730	−3·070	−5·340	−2·450
R^2^	0·07	0·14	0·13	0·10	0·15	0·05
F-statistic	3·06	6·86	6·35	4·76	7·52	2·09

Unsat. fat, unsaturated fat; Sat. fat, saturated fat; Carbo, carbohydrates.

* *P* < 0·05,

** *P* < 0·01,

*** *P* < 0·001.

Only variables of interest are displayed here. Full regression tables are shown in Supplementary Tables S11 and S12. t-Ratios are based on robust standard errors. For F-statistic of the null hypothesis, all coefficients are equal to zero.

*n* = 261.

Notably, socio-economic characteristics are poor predictors of dietary behaviour following a T2D diagnosis. With few exceptions, there are no statistically significant differences in dietary change following a diagnosis between the included socio-economic characteristics. Full results are presented in Supplementary Tables S11 and S12. As could be expected, diagnosed individuals who are in single households show a larger improvement in overall healthiness (HEI). This does not necessarily imply that single-household individuals are more successful in improving their diets. If diagnosed individuals in larger households make changes in their diet, while the rest of the household does not make such changes, the dietary changes measured on the household basis are less clear compared to a single household.

Contrary to the socio-economic characteristics, the baseline diet is an important explanatory variable for the change in diet following diagnosis. Individuals with a relatively poor HEI prior to their diagnosis improve their HEI compared to those with a higher HEI prior to diagnosis. In other words, individuals with relatively poor overall diet prior to diagnosis changed more towards a healthy diet.

The pattern that baseline consumption is correlated with changes in consumption following diagnosis holds for most food and nutrition categories. For example, individuals with a relatively high share of saturated fat in their consumption reduced their energy percentage from saturated fat more compared to those individuals with a lower baseline consumption. Similar conclusions hold for unsaturated fat and added sugar; individuals in households with high shares from these nutrients decrease their percentages from these nutrients compared with the central group. The HEI show that largest improvements are found for those with a lower HEI value, which might have the natural interpretation that those with the worst diet have more to improve to follow the recommendations. For meat, we see that those who have a larger energy share in the first place decrease consumption most, and the same holds for the unhealthy food groups, SSB, cake and candy.

### Robustness in findings

In our analysis, consumption measures for specific food and nutrient categories are expressed as percentages of total energy consumption. An individual whose diet consists of a high share of saturated fat may have adjusted their diet by reducing the total saturated fat consumption. Even if the individual makes no other changes to the diet, the energy share for the rest of the categories are then affected. The energy share from sugar will increase, even if the total amount of sugar consumed is unchanged. For robustness, we therefore re-estimate all models presented in Fig. 1 (equation 1), while measuring the outcome variables in absolute energy intake rather than in energy shares. Results from these models are presented in Supplementary Table S1. Overall, the main results from these models are similar. Fruit and vegetable consumption increases, while meat and fish consumption decreases in the long term. While there are no significant long-term effects on the unhealthy products (SSB, candy and cakes) when measured in shares, there are significant increases in the total energy from these food groups. Further, added sugar and carbohydrates increase in the long term for both share and total energy. For protein and fat (unsaturated and saturated), only the total energy displays statistically significant long-term changes. Interestingly, we find that total energy consumed decrease significantly.

Moreover, we also re-estimate the models presented in Table 2, where the dependent variable is the change following diagnosis (equation 3) with the real changes instead of the changes in energy shares. Results from these estimations are presented in Supplementary Table S2. For the nutrients, those with a high consumption of protein, unsaturated and saturated fat as well as fibre and added sugar decrease consumption the most. For the unhealthy foods, such as SSB and candy, we see the same tendency. Hence, the main conclusion is unchanged; larger room is found for improvement in the diet of those with the most unhealthy diet and no effects are found from sociodemographic variables. There are some minor differences in the results compared to the estimations in shares. These can be explained by the fact that we find that total energy consumed decreases most for those with largest energy consumption prior to diagnosis of T2D.

The identification of T2D diagnosis accommodates individuals who have been diagnosed at a hospital and/or had a complication related to T2D, treated at a hospital. We further use first occurrence of prescribed T2D medicine as identification of a diagnosis for individuals treated with T2D medicine, but without a diagnosis or complication registered in our data. For robustness, we estimate the models presented in Fig. 1 (and Table A2) based on a stricter identification of diagnosis. We then only include individuals who are diagnosed or have had a complication, such that individuals who are identified based on their medical prescription are excluded. We thereby test the results if they are sensitive to potential inclusion of individuals treated with antihyperglucemic drugs for other diagnoses than T2D (e.g. polycystic ovarian syndrome, gestational diabetes mellitus and type 1 diabetes). This gives a smaller sample of T2D individuals (*n* 60 *v*. *n* 274). Overall, the results from this smaller sample provide similar results, with fewer statistically significant estimates, which is to be expected given the smaller sample size. The results for this stricter definition are provided in Supplementary Table S3. We also re-estimated equation 3 with this stricter definition, but due to the small sample size most parameters were insignificant. We have omitted these results from the material.

One limitation of the study is that we have purchase data on the household level, while the diagnosis data are on the individual level. This implies that there may be diagnosed individuals living in a household with someone who continue to eat as before. To test the robustness of our results to the results that might appear from some kind of household bargaining over diets, we re-estimate the results from Table A2 and Table A3 with only single households. The results of these estimations are shown in Supplementary Table S4. Main results are unchanged. We also re-estimated equation 3 with only singles, but since we only have eighty individuals, the majority of parameters are insignificant whereas this is omitted from the supplementary material.

To test if the diagnosis variable in our regressions in Table A2 actually measures an effect of a diagnosis, we estimate a set of regressions for robustness, where we include lead variables for the diagnosis. Hence, a variable indicating the months prior to the diagnosis is included in equation 1. If the diagnosis effect in equation 1 is indeed the effect of a diagnosis, the lead variable should be zero (there should not be an effect on diet prior to the diagnosis). We estimate regressions including 1-, 2-, 3- and 6-month lead variables. We present the results in Supplementary Tables S5–S8. For all models, the lead variables are statistically insignificant.

A main assumption in the event study analysis is that of parallel trends, implying that the diagnosed individuals follow the same trend as the undiagnosed individuals prior to their diagnosis. To test if this assumption holds, we re-estimate equation 1 (displayed in Fig. 1 and Table A2) while including interaction terms between the time variables (year and month dummies) and the diagnosed individuals in the time periods prior to their diagnosis. If the parallel trend assumption holds, the interactions between the time dummies and the diagnosed individuals prior to their diagnosis should be zero. The results support the parallel trend assumption, as revealed in the last row of Supplementary Table S9 for some models, but not for all. We therefore re-estimate equation 1 with only the diagnosed individuals (before and after their diagnosis). Results are displayed in Supplementary Table S10.

## Discussion

We explore the dietary effects following T2D diagnosis by combining several high-quality data sources. First, we include detailed medical records, as registered by the official national registry in Denmark. Diagnosis is identified based on either diagnosis, treatment of complication related to T2D or medical prescription for treating T2D. These data are merged with data on individuals’ socio-economic characteristics. Finally, we combine these data with the individuals’ household food purchase data, as registered in a consumer panel where all purchases are scanned and reported on a daily basis. Our sample includes 274 individuals who are diagnosed with T2D while reporting food purchases in the consumer panel. We have additional data on 15 099 individuals not identified with T2D. Event study regression analysis indicates some changes in dietary composition following diagnosis. In particular, at the positive side, we see an immediate increase in the overall dietary healthiness, as measured by a HEI, increase in fruit and vegetable consumption, and decrease in meat consumption, saturated fat as well as candy and cake consumption. However, we also see increase in consumption of added sugar, and the majority of the positive immediate changes are reversed when we consider the 12 months ahead. Our main findings are hereby consistent with findings from other studies, which suggest that diagnosis on lifestyle-related diseases have limited impact on food consumption^(32)^. Our results hold across a range of robustness tests.

We further explore heterogeneity in dietary changes following diagnosis. Two main conclusions can be drawn from this analysis. First, socio-economic characteristics are poor predictors of dietary changes following diagnosis. Evidence from studies on general populations suggest that responses to public health information vary with gender and income levels. However, we do not find differences in dietary change between men and women, or between income levels. These findings are supported by Oster^(32)^, who concludes that personal characteristics are not strongly correlated with dietary changes following a diagnosis. Our findings suggest that behavioural changes based on health promotion in the general population cannot be generalised to T2D individuals, as these are not a random sample of the population.

Second, changes in purchase patterns following diagnosis vary with the pre-diagnosis consumption patterns. In general, individuals with relatively poor diet prior to diagnosis make relatively more health-beneficial changes compared to individuals with a relatively healthier diet prior to the diagnosis. A possible explanation to this pattern could be that these individuals have more room for improvement as pre-diagnosis consumption levels are lower, an explanation in line with Oster^(32)^. It is also possible that individuals with healthier purchase patterns prior to a diagnosis make other types of changes such as quitting smoking, being more physically active or consuming less alcohol. It may also be that those with relatively healthier diet are less interested in participating in the offered nutrition support and education. The latter explanation might be an interesting route for further research. On the positive note, we find that for unhealthy nutrients and foods also that those with the largest pre-diagnosis level of consumption have the largest decrease in consumption.

A central strength with this study compared to previous studies is that we use registry data. Existing evidence on the impact of a T2D diagnosis on food consumption is based on self-reported diagnosis status^(31–34)^. In addition, detailed results are available in this study on food purchases, as reported daily in a consumer panel. This has the advantage compared to self-reported dietary intake measurements^(34,36,37)^, where memory and selective reporting are potential sources of measurement error.

We note that individuals, who are diagnosed by their GP with no medical prescriptions or any treated complications, are not identified as T2D individuals in our data. These individuals may make the largest dietary improvements, since they are successful in avoiding complications and medication. If so, the estimated changes in diet in our sample are downward-biased; the true average effects from a diagnosis are larger than we see.

### Limitations

While the quality of the combined data sources used in this study is an important strength compared to previous studies, some potential misclassifications of the diagnosis and food purchases must be taken into consideration when interpreting the results.

First, while participants in the consumer panel are asked to report all their daily purchases, some purchases may be more likely to be underreported. It can be expected that unhealthy products, such as spontaneous purchases of snacks and sweets, are un- or under-reported. Such misreporting is mainly a concern if the likelihood of misreporting is affected by a diagnosis. We do not have reasons to expect this, but it should be considered an uncertainty when interpreting our results.

Further, the reporting of purchases is made on a household basis. We assume that the distribution of the purchased food is even, based on standard individual measures which were constructed based on recommended nutrient requirements. If the consumption distribution changes in the household following the diagnosis of an individual, this is a source of bias. However, we do not have any expectations that this takes place in a systematic way. Finally, while our data sources are of high quality, the sample of individuals included in this study is limited (274). Despite these limitations, our study contributes with insights based on reliable data sources.

Another limitation with the present study is that we cannot disentangle the effect of the information from the effect of a diagnosis itself and the effect of dietary guidance offered to the diagnosed individual. Unfortunately, our data do not indicate if the individual accepted the offer of dietary guidance by dieticians. What we do know is that a diagnosis made in conjunction with a hospital visit, which is the diagnosis that is included in our data, is always accompanied by an offer to obtain professional dietarian guidance. Those that have mild T2D will often get the offer via their own GP and possibly a referral to the municipality. Those with more complicated T2D will in many cases be referred to diabetes control by a specialist at a hospital or a T2D centre and receive dietitian guidance there. Thus, in this study, we test the effect of a diagnosis itself and cannot control if additional dietary support occurs.

### Implications for policy and future research

The main finding in this study is that a diagnosis of the lifestyle-related disease T2D has limited impact on food consumption. The low dietary adherence following a diagnosis might partially be explained by the fact that those who are able to change their dietary behaviour have already done so since body weight management, treatment of high cholesterol levels and hypertension are often preceding a T2D diagnoses. Hence, the diagnosed individuals are not a random sample of the population, but mainly self-selected individuals who have failed to react to earlier warning messages. If possible, future studies should compare the socio-economic profiles of those reacting to these pre-warnings with the general panel and the diabetic sample.

Importantly, our findings emphasise the difficulty for diagnosed individuals to change their dietary habits, especially on a long-term basis. Hence, the low adherence to dietary guidelines following a T2D underlines the need for additional measures and policies to support and induce dietary improvements among diagnosed individuals, preferably some months after diagnosis to maintain healthy dietary changes. More research is needed to identify successful methods for supporting T2D diagnosed individuals in changing food consumption in line with dietary guidelines.

Another area that future research should investigate is the effects of medication. If the diet is changed in accordance with the guidelines, the need for T2D medication may be significantly reduced or postponed. However, the use of traditional medication reduces the symptoms even without dietary changes and newer medication types (GLP1 receptor agonists) help to regulate food intake, increasing preferences for healthier diets. An important question is if traditional pharmaceutical treatment actually induces worse dietary behaviour as it reduces the present utility costs of disease by reducing some of the potential disease symptoms associated with having a poor diet in the short run and if newer treatment varieties actually lead to improvements in dietary quality. Future research should explore these relationships.

## Supplementary material

For supplementary material accompanying this paper visit https://doi.org/10.1017/S1368980022001409. click here to view supplementary material
