# Supplementary material for: Dietary changes based on food purchase patterns following a type 2 diabetes diagnosis
Source: Public Health Nutr. 2022 Jun 17;25(10):2782–93. doi: 10.1017/S1368980022001409 (PMC9991834; doi:10.1017/S1368980022001409)
Supplement: Supplementary file 1 [file S1368980022001409sup.zip › S1368980022001409sup001.docx]

## Appendix

### A1. Healthy Eating Index

The HEI index is composed by scores, quantifying how well each household follows the official dietary recommendations. Each of the eight dietary recommendation gives the household a score between 0 and 1, 0 being the worst possible score and 1 if the household fulfil the dietary recommendation. Scores are estimated based on energy shares as follows:

$$Score_{int}=\left( \frac{s_{int}-s_{0,i}}{s_{recommended,ni}-s_{0,i}} \right)$$

Where s_int_ is the n’^th^ households’ consumption of the given food or nutrient *i* at the time *t*, s_recommended,ni_ is the recommended consumption and s_0,i_ is the consumption level that will yield a score of zero. Defining the consumption level for s_0_ is based on the worst level of consumption observed in data. The linearity indirectly assumed in the measure is a weakness. In reality, the marginal value for dietary quality for a piece of fruit might be greater if the initial consumption is zero compared if the initial consumption is three. The model does not encounter such differences.

Having obtained the scores for each of the eight dietary recommendations investigated, the scores for each household are weighted together yielding one HEI value for each household *n* at time *t*. When pooling the scores into the HEI, no weights are applied, meaning that all dietary recommendations are considered equally important for a healthy diet. This might not be completely appropriate since some recommendations might have a greater impact on dietary quality but identifying and justifying any differentiating in scores by ranking the dietary recommendations is not within the scope of this analysis. The individual scores are weighted together using a Euclidean distance measure. There are eight components in the HEI measure, denoted by *i, n* is household identification number and *t* is time:

$$HEI_{nt}= \sqrt{\sum_{1=i}^{8} \left( score_{int} \right)^{2}}$$

The original HEI runs from 0 to $\sqrt{8}$ and is rescaled to run from 0 to 100, for ease of interpretation.

Table A1. Operationalization of the official 2013 Danish dietary guidelines for Healthy Eating Index (HEI).

| Food or nutrient category | Official recommendation | Recommended pct. of daily calorie intake |
| --- | --- | --- |
| Fruit and vegetables | Min. 600 g per day, hereof at least 300g vegetables (300g-500g for children between 4-10years) | Min. 11.5 pct.^1^ |
| Fish | Min. 350 g per week | Min. 3.5 pct.^2^ |
| Wholegrain | Min. 75 g per day (Min 25-35 g dietary fibre) per day | Min. 2.4 pct. |
| Meat from beef, veal, lamb or pork | Max 500 g per week | Max. 7 pct.^3^ |
| Fat | Max. 25-40 pct. of the daily calorie intake | Max. 25-40 pct. |
| Saturated fats | Max. 10 pct. of the daily calorie intake. | Max. 10 pct. |
| Added sugar | Max. 10 pct. of the daily calorie intake. | Max. 10 pct. |
| Salt | Max 6-7 g daily |  |

Source: Source: The Danish Veterinary and Food Administration: [www.altomkost.dk](http://altomkost.dk/deofficielleanbefalingertilensundlivsstil/de-officielle-kostraad/) , accessed November 2020

1: The number shown in the table is calculated as the average energy density for fruit and vegetables in the purchase data (1.96 kJ/g) multiplied by the recommended intake per day and divided by the average recommended total calorie intake in kJ per day per person (7970kJ for kids 2-17 years and 9995 kJ for adults). In the specific HEI calculations, household-specific energy densities according to type of fruit and vegetable consumed in the household are used together with approximate energy requirements based on household composition.

2: The number shown in the table is calculated as the average energy density for fish in the purchase data (6.80 kJ/g) multiplied by the recommended 300g per week (≈43g. per day) and divided by the average recommended total calorie intake in kJ per day per person (7970kJ for kids 2-17 years and 9995 kJ for adults). In the specific HEI calculations, household-specific energy densities according to type of fish consumed in the household are used together with approximate energy requirements based on household composition.

3: The number shown in the table is calculated as the average energy density for meat in the purchase data (9.65 kJ/g) multiplied by the recommended 500g per week (≈71g. per day) and divided by the average recommended total calorie intake in kJ per day per person (7970kJ for kids 2-17 years and 9995 kJ for adults 18-64). In the specific HEI calculations, household-specific energy densities according to type of fish consumed in the household are used together with approximate energy requirements based on household composition.

Table A2. Changes in dietary consumption after a T2D diagnosis

|  | Overall diet healthiness | Energy share from food groups | | | | | | | |  |
| --- | --- | --- | --- | --- | --- | --- | --- | --- | --- | --- |
|  | HEI |  | Fruit & vegetables | Fish | Meat |  | SSB^c^ | Cakes | Candy | |
| Diabetes | 1.06*** |  | 1.09*** | 0.08 | -0.77*** |  | 0.06 | -0.11 | -0.21* | |
| \|t-ratio\| | 5.64 |  | 5.52 | 1.18 | 3.55 |  | 0.59 | 1.09 | 2.31 | |
| Diabetes 12m | -1.40*** |  | -0.53** | -0.29*** | 0.29 |  | -0.06 | 0.22* | 0.32*** | |
| \|t-ratio\| | 7.62 |  | 2.87 | 3.97 | 1.35 |  | 0.62 | 2.31 | 3.87 | |
| R^2^ | 0.40 |  | 0.40 | 0.29 | 0.33 |  | 0.33 | 0.30 | 0.34 | |
| P-all zero^a^ | <0.001 |  | <0.001 | <0.001 | <0.001 |  | <0.001 | <0.001 | <0.001 | |
| P-diabetes^b^ | 0.027 |  | <0.001 | <0.001 | 0.006 |  | 0.964 | 0.119 | 0.132 | |

Notes: t-ratios are based on robust standard errors. *** p < 0.001, ** p < 0.01, * p < 0.05.

Models are estimated as specified in eq (1). All models include a constant, year-dummies, month-dummies and individual fixed effects.

Number of observations=366 036 Number of individuals=6 430

^a^P-all zero refers to F-test of the null hypothesis that all coefficients are equal to zero.

^b^P-diabetes refers to F-test of the null hypothesis that *Diabetes* + *Diabetes12m* = 0

^c^ Sugar sweetened beverages.

Table A2. continued

|  |  | | Energy share from nutrients | | | | | |
| --- | --- | --- | --- | --- | --- | --- | --- | --- |
|  |  | Protein | | Unsaturated fat | Saturated fat | Added sugar | Carbo-hydrates | Fiber |
| Diabetes |  | 0.31*** | | -0.22 | -0.59*** | 0.35*** | 0.78** | 0.09*** |
| \|t-ratio\| |  | 3.22 | | 1.47 | 4.88 | 3.71 | 3.11 | 4.20 |
| Diabetes 12m |  | -0.25** | | 0.13 | 0.68*** | 0.07 | -0.18 | -0.07*** |
| \|t-ratio\| |  | 2.63 | | 0.87 | 5.74 | 0.73 | 0.76 | 3.47 |
| R^2^ |  | 0.31 | | 0.21 | 0.29 | 0.25 | 0.32 | 0.33 |
| P-all zero^a^ |  | <0.001 | | <0.001 | <0.001 | <0.001 | <0.001 | <0.001 |
| P-diabetes^b^ |  | 0.448 | | 0.416 | 0.335 | <0.001 | 0.003 | 0.316 |

Notes: t-ratios are based on robust standard errors. *** p < 0.001, ** p < 0.01, * p < 0.05.

Models are estimated as specified in eq (1). All models include a constant, year-dummies, month-dummies and individual fixed effects.

Number of observations=366 036 Number of individuals=6 430

^a^P-all zero refers to F-test of the null hypothesis that all coefficients are equal to zero.

^b^P-diabetes refers to F-test of the null hypothesis that *Diabetes* + *Diabetes12m* = 0

### Table A3. Change in dietary measures six months before and six months after diagnosis

|  | Mean | Std. Dev. | Median | 5% | 95% |
| --- | --- | --- | --- | --- | --- |
| Overall healthiness |  |  |  |  |  |
| HEI | 1.09 | 5.47 | 1.15 | -7.63 | 9.77 |
| Energy from food categories |  |  |  |  |  |
| Fruit and vegetable | 0.95 | 6.53 | 0.22 | -4.86 | 6.40 |
| Fish | 0.20 | 1.71 | 0.00 | -1.57 | 2.70 |
| Meat | -0.43 | 6.13 | -0.55 | -10.92 | 9.13 |
| SSB | -0.11 | 3.06 | 0.00 | -4.06 | 3.53 |
| Cakes | 0.01 | 3.05 | 0.00 | -3.35 | 3.61 |
| Candy | -0.25 | 2.67 | 0.25 | -4.08 | 2.53 |
| Energy from nutrients |  |  |  |  |  |
| Protein | 0.17 | 2.65 | 0.16 | -4.11 | 3.76 |
| Unsaturated fat | -0.22 | 3.28 | -0.70 | -5.87 | 5.87 |
| Saturated fat | -0.69 | 3.28 | -0.70 | -5.61 | 4.63 |
| Added sugar | 0.34 | 2.97 | 0.00 | -3.26 | 4.08 |
| Carbohydrates | 0.69 | 6.16 | 0.04 | -9.67 | 9.71 |
| Fibre | 0.07 | 0.59 | 0.04 | -0.81 | 1.05 |

Note: N=264. The changes for the product categories and nutrients are in percentage points.

| **Figure A1: Scatter plot with the dietary change plotted on pre-diagnoses consumption level** | |
| --- | --- |
| 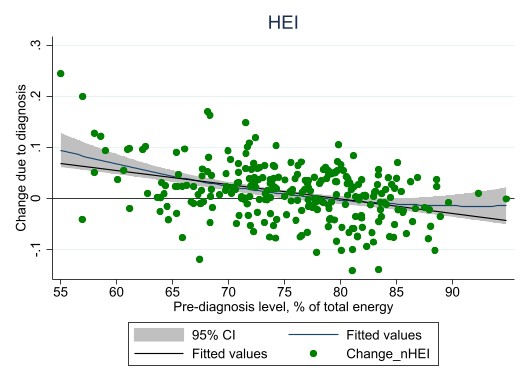 | 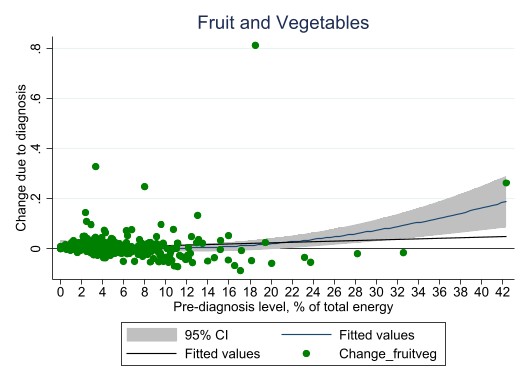 |
| 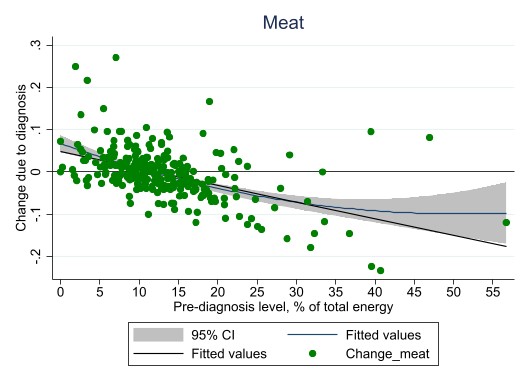 | 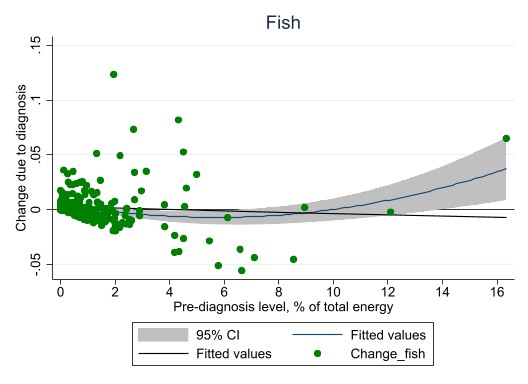 |
| 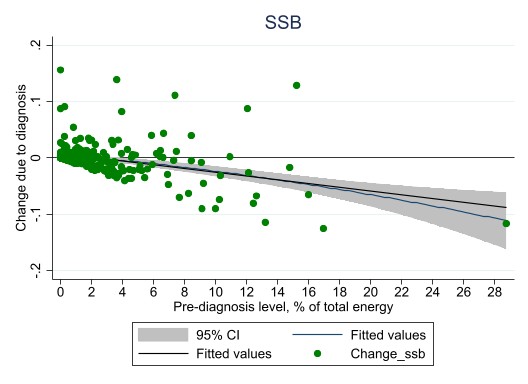 | 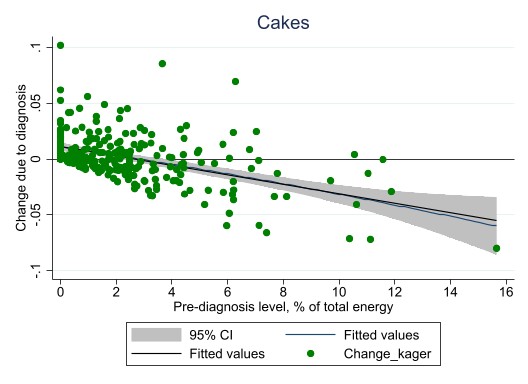 |
| 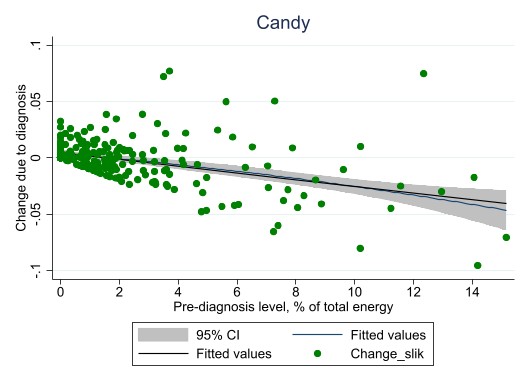 | 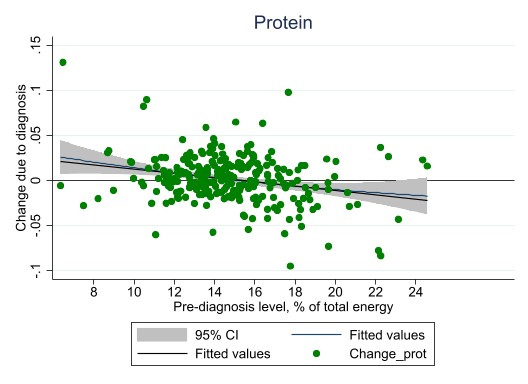 |
| 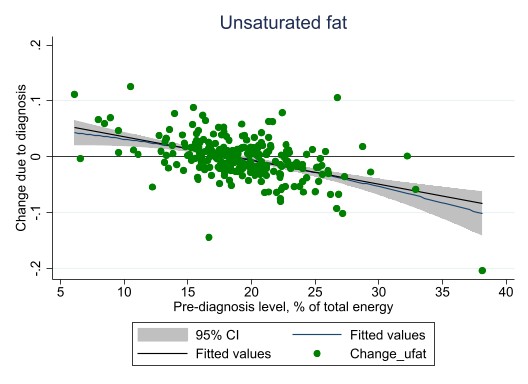 | 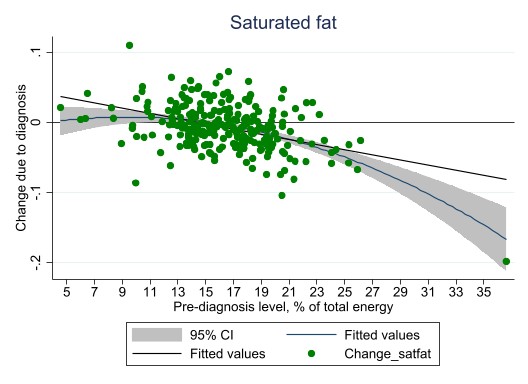 |
| 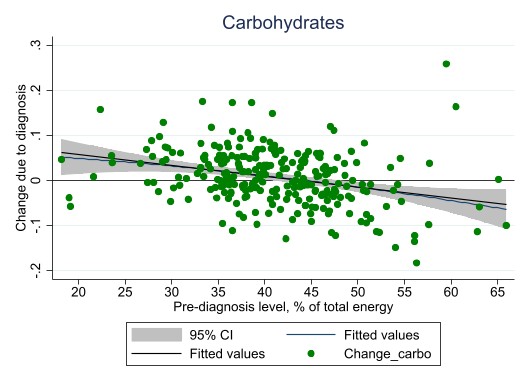 | 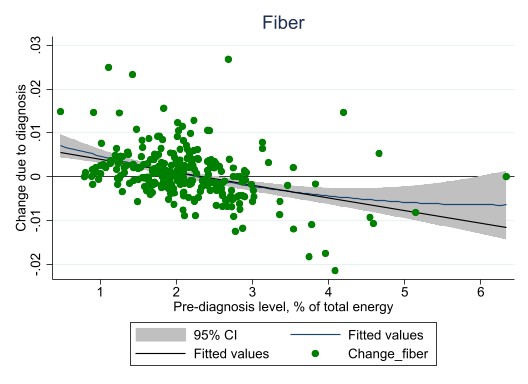 |
| 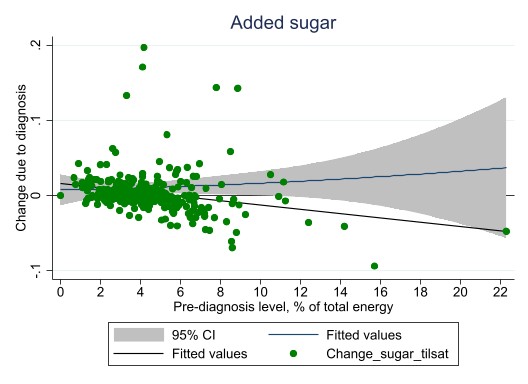 |  |
| Notes: Simple linear and quadratic tendencies are plotted on the observed data to illustrate the correlation | |
