# Supplementary material for: Dietary changes based on food purchase patterns following a type 2 diabetes diagnosis
Source: Public Health Nutr. 2022 Jun 17;25(10):2782–93. doi: 10.1017/S1368980022001409 (PMC9991834; doi:10.1017/S1368980022001409)
Supplement: Supplementary file 1 [file S1368980022001409sup.zip › S1368980022001409sup002.docx]

Supplementary Material

Table S1. Changes in dietary consumption after a T2D diagnosis. Total energy (KJ consumed per month).

|  |  |  |  | Energy from food groups | | | |  | | Energy from unhealthy food groups | | |
| --- | --- | --- | --- | --- | --- | --- | --- | --- | --- | --- | --- | --- |
|  | Total energy |  |  | Fruit & vegetables | Fish | Meat |  | | SSB^c^ | | Cakes | Candy |
| Diabetes | -67762* |  |  | 781 | 160 | -3216*** |  | | 376 | | -330 | -671* |
| \|t-ratio\| | 2.07 |  |  | 1.40 | 0.62 | 3.91 |  | | 1.28 | | 1.27 | 2.30 |
| Diabetes 12m | 19756*** |  |  | 955 | -671* | 1469 |  | | 266 | | 1193*** | 1434*** |
| \|t-ratio\| | 5.79 |  |  | 1.38 | 2.12 | 1.75 |  | | 0.90 | | 4.55 | 5.15 |
| Constant | 292442*** |  |  | 20704*** | 72033 | 36940*** |  | | 3340*** | | 3964* | 360 |
| \|t-ratio\| | 4.00 |  |  | 11.81 | 1.13 | 6.88 |  | | 5.26 | | 2.24 | 0.39 |
| Observations | 366036 |  |  | 351423 | 366036 | 345307 |  | | 366036 | | 366036 | 366036 |
| Individuals | 6430 |  |  | 6366 | 6430 | 6344 |  | | 6430 | | 6430 | 6430 |
| R^2^ | 0.60 |  |  | 0.14 | 0.33 | 0.41 |  | | 0.29 | | 0.42 | 0.35 |
| P-all zero^a^ | <0.001 |  |  | <0.001 | <0.001 | <0.001 | | | <0.001 | | <0.001 | <0.001 |
| P-diabetes^b^ | <0.001 |  |  | 0.025 | 0.002 | 0.014 |  | | 0.014 | | <0.001 | 0.007 |
| Note: t-ratios are based on robust standard errors. *** p < 0.001, ** p < 0.01, * p < 0.05. Models are estimated as specified in eq (1). All models include year-dummies, month-dummies and individual fixed effects. ^a^P-all zero refers to F-test of the null hypothesis that all coefficients are equal to zero. .  ^b^P-diabetes refers to F-test of the null hypothesis that *Diabetes* + *Diabetes12m* = 0 , ^c^ Sugar sweetened beverages. | | | | | | | | | | | | |

Table S1. Cont.

|  |  |  | | Energy from nutrients | | | | | |  |
| --- | --- | --- | --- | --- | --- | --- | --- | --- | --- | --- |
|  |  |  | Protein | | Unsaturated fat | Saturated fat | Added sugar | Carbohydrates | Fiber |  |
| Diabetes |  |  | 160 | | -33 | -53*** | 37 | -15 | 2 |  |
| \|t-ratio\| |  |  | 0.60 | | 1.67 | 3.32 | 1.85 | 0.15 | 0.19 |  |
| Diabetes 12m |  |  | 89** | | 88*** | 112*** | 104*** | 547*** | 37** |  |
| \|t-ratio\| |  |  | 3.21 | | 4.47 | 7.22 | 4.12 | 5.08 | 3.14 |  |
| Constant |  |  | 2143 | | 2027 | 887 | 522 | 5935 | 651 |  |
| \|t-ratio\| |  |  | 18.59 | | 2.08 | 10.23 | 7.05 | 9.16 | 12.71 |  |
| Observations |  |  | 366036 | | 366036 | 366036 | 366036 | 366036 | 366018 |  |
| Individuals |  |  | 6430 | | 6430 | 6430 | 6430 | 6430 | 6430 |  |
| R^2^ |  |  | 0.60 | | 0.55 | 0.58 | 0.21 | 0.51 | 0.44 |  |
| P-all zero^a^ |  |  | <0.001 | | <0.001 | <0.001 | <0.001 | <0.001 | <0.001 |  |
| P-diabetes^b^ |  |  | <0.001 | | 0.002 | <0.001 | <0.001 | <0.001 | <0.001 |  |
| Note: t-ratios are based on robust standard errors. *** p < 0.001, ** p < 0.01, * p < 0.05. Models are estimated as specified in eq (1). All models year-dummies, month-dummies and individual fixed effects ^a^P-all zero refers to F-test of the null hypothesis that all coefficients are equal to zero.  ^b^P-diabetes refers to F-test of the null hypothesis that *Diabetes* + *Diabetes12m* = 0. | | | | | | | | | |  |

Table S2. Change in diet six months after diagnosis compared to six months prior to diagnosis. Total energy.

|  | Overall Energy |  | Energy from food groups | | |  | Energy from unhealthy food groups | | | | |
| --- | --- | --- | --- | --- | --- | --- | --- | --- | --- | --- | --- |
|  |  |  | Fruit & veg. | Fish | Meat |  | SSB^b^ | Cakes | Candy | |  |
| Female | 9448 |  | 3304^***^ | -251 | 4459 |  | -1345 | 186 | 402 | |  |
| (\|t- ratio\|) | 0.86 |  | 2.62 | -0.39 | 0.95 |  | -1.23 | 0.25 | 0.47 | |  |
| Single household | 1256 |  | 924 | 614 | 2741 |  | 16695 | 1105 | 375 | |  |
| (\|t- ratio\|) | 0.11 |  | 0.69 | 0.90 | 0.55 |  | 1.41 | 1.40 | 0.41 | |  |
| Age | -487 |  | 380 | -30 | 85 |  | -370^*^ | -98 | -245 | |  |
| (\|t- ratio\|) | -0.22 |  | 1.49 | -0.23 | 0.09 |  | -1.68 | -0.66 | -1.41 | |  |
| Income | 7121 |  | 24895 | 42264^***^ | 17180 |  | 23637 | -2216 | -8223 | |  |
| (\|t- ratio\|) | 0.03 |  | 1.00 | 3.26 | 0.19 |  | 1.10 | -0.15 | -0.49 | |  |
| Age^2^ | 10.71 |  | -3.01 | 0.46 | 1.94 |  | 3.15 | 1.12 | 2.18 | |  |
| (\|t- ratio\|) | 0.54 |  | -1.34 | 0.39 | 0.23 |  | 1.62 | 0.85 | 1.42 | |  |
| Income^2^ | 92 |  | -997 | -1688^***^ | -708 |  | -979 | 119 | 3702 | |  |
| (\|t- ratio\|) | 0.01 |  | -0.97 | -3.14 | -0.18 |  | -1.10 | 0.20 | 0.53 | |  |
| Baseline | -0.14^***^ |  | 0.01 | -0.66^***^ | -0.16 |  | -0.20^**^ | -0.21^***^ | -0.34^***^ | |  |
| (\|t- ratio\|) | -2.45 |  | 0.09 | -6.06 | -1.64 |  | -2.12 | -2.93 | -4.32 | |  |
| Baseline^2^ | -5.8E-08^***^ |  | -6.4E-06^***^ | 2.1E-05^***^ | -1.8E-07 |  | -2.3E-06 | -5.3E-06^***^ | 3.4E-07 | |  |
| (\|t- ratio\|) | -2.79 |  | -2.58 | 8.58 | -1.13 |  | -1.40 | -4.36 | 0.23 | |  |
| Constant | -69057 |  | -165073^***^ | -262102 | -112006 |  | -130325 | 12662 | 527515 | |  |
| (\|t- ratio\|) | -0.20 |  | -3.37 |  | -1.11 |  | -1.01 | 0.15 | 0.52 | |  |
| Observations | 261 |  | 258 | 261 | 258 |  | 261 | 261 | 261 | |  |
| R2 | 0.36 |  | 0.19 | 0.27 | 0.14 |  | 0.17 | 0.40 | 0.25 | |  |
| F-statistic^a^ | 17.91 |  | 7.19 | 11.40 | 5.20 |  | 6.40 | 21.15 | 10.72 | |  |
| Note: Income is included in logarithmic form. T-ratios are based on robust standard errors. ^a^F-statistic of the null hypothesis that all coefficients are equal to zero. ^b^SSB = Sugar Sweetened Beverages. *** p < 0.001, ** p < 0.01, * p < 0.05. N=261 | | | | | | | | | |  |  |

Table S2 Cont.

|  |  | Energy from nutrients | | | | | | | | | |
| --- | --- | --- | --- | --- | --- | --- | --- | --- | --- | --- | --- |
|  |  | Protein | Unsaturated fat | | Saturated fat | | Added sugar | | Carbohydrates | | Fiber |
| Female |  | 158.11 | 11.42 | 50.27 | | 25.44 | | 286.35 | | 47.79 | |
| (\|t- ratio\|) |  | 1.62 | 0.18 | 0.90 | | 0.43 | | 0.87 | | 1.60 | |
| Single household |  | 26.68 | -2.71 | -16.42 | | 90.97 | | 120.68 | | 55.20^*^ | |
| (\|t- ratio\|) |  | 0.26 | -0.04 | -0.27 | | 1.43 | | 0.34 | | 1.74 | |
| Age |  | 6.18 | -0.37 | -0.55 | | 5.04 | | -37.90 | | 6.77 | |
| (\|t- ratio\|) |  | 0.31 | -0.03 | -0.05 | | 0.42 | | -0.57 | | 1.11 | |
| Income |  | 960.44 | 466.29 | -158.54 | | -83.68 | | 357.29 | | -419.40 | |
| (\|t- ratio\|) |  | 0.50 | 0.36 | -0.14 | | -0.07 | | 0.05 | | -0.71 | |
| Age^2^ |  | 0.01 | 0.03 | 0.04 | | -0.05 | | 0.48 | | -0.05 | |
| (\|t- ratio\|) |  | 0.03 | 0.24 | 0.36 | | -0.48 | | 0.82 | | -0.90 | |
| Income^2^ |  | -37.41 | -17.52 | 8.02 | | 3.22 | | -9.33 | | 17.92 | |
| (\|t- ratio\|) |  | -0.47 | -0.33 | 0.17 | | 0.07 | | -0.03 | | 0.73 | |
| Baseline |  | -0.20^***^ | -0.20^***^ | -0.21^***^ | | -0.32^***^ | | -0.14^**^ | | -0.15^***^ | |
| (\|t- ratio\|) |  | -3.46 | -3.33 | -3.23 | | -3.86 | | -2.24 | | -2.88 | |
| Baseline^2^ |  | -2.7E-06 | -1.5E-05^***^ | -2.0E-05^***^ | | -1.5E-06^**^ | | -1.4E-05 | | -2.7E-06^***^ | |
| (\|t- ratio\|) |  | -1.37 | -3.05 | -2.95 | | -2.16 | | -1.54 | | -2.61 | |
| Constant |  | -6092.14 | -2819.62 | 880.56 | | 672.98 | | -1532.09 | | 2313.17 | |
| (\|t- ratio\|) |  | -0.53 | -0.37 | 0.13 | | 0.10 | | -0.04 | | 0.65 | |
| Observations |  | 261 | 261 | 261 | | 261 | | 261 | | 261 | |
| R2 |  | 0.37 | 0.41 | 0.38 | | 0.33 | | 0.21 | | 0.44 | |
| F-statistic^a^ |  | 18.75 | 21.76 | 19.11 | | 15.76 | | 8.51 | | 25.17 | |
| Note: Income is included in logarithmic form. T-ratios are based on robust standard errors. ^a^F-statistic of the null hypothesis that all coefficients are equal to zero. *** p < 0.001, ** p < 0.01, * p < 0.05. N=261 | | | | | | | | | | | |

Table S3. Changes in dietary consumption after a T2D diagnosis (strict definition, medicine identification excluded)

|  | Overall diet healthiness |  | Energy share from food groups | | | |  | | Energy share from unhealthy food groups | | |  |
| --- | --- | --- | --- | --- | --- | --- | --- | --- | --- | --- | --- | --- |
|  | HEI |  | Fruit & vegetables | Fish | Meat |  | | SSB^c^ | | Cakes | Candy | |
| Diabetes | 1.01*** |  | 0.01 | 0.15 | -0.72* |  | | 0.29 | | -0.02 | -0.01 | |
| \|t-ratio\| | 3.49 |  | 0.05 | 1.51 | -2.02 |  | | 1.34 | | 0.12 | 0.07 | |
| Diabetes 12m | -0.84** |  | -0.05 | -0.20* | 0.17 |  | | -0.01 | | 0.08 | 0.18 | |
| \|t-ratio\| | 3.06 |  | 0.23 | -1.97 | 0.49 |  | | 0.06 | | 0.42 | 1.20 | |
| R^2^ | 0.40 |  | 0.41 | 0.29 | 0.33 |  | | 0.33 | | 0.30 | 0.33 | |
| P-all zero^a^ | <0.001 |  | <0.001 | <0.001 | <0.001 |  | | <0.001 | | <0.001 | <0.001 | |
| P-diabetes^b^ | 0.506 |  | 0.864 | 0.511 | 0.082 |  | | 0.096 | | 0.619 | 0.186 | |
| Note: t-ratios are based on robust standard errors. *** p < 0.001, ** p < 0.01, * p < 0.05. Models are estimated as specified in eq (1). All models include a constant, year-dummies, month-dummies and individual fixed effects. Number of observations=350 325 Number of individuals=6 266 ^a^P-all zero refers to F-test of the null hypothesis that all coefficients are equal to zero. ^b^P-diabetes refers to F-test of the null hypothesis that *Diabetes* + *Diabetes12m* = 0 ^a^ Sugar sweetened beverages. ^c^SSB = Sugar Sweetened Beverages | | | | | | | | | | | | |

Table S3. Cont.

|  |  |  | | Energy from nutrients | | | | | |
| --- | --- | --- | --- | --- | --- | --- | --- | --- | --- |
|  |  |  | Protein | | Unsaturated fat | Saturated fat | Added sugar | Carbohydrates | Fiber |
| Diabetes |  |  | 0.17 | | -0.26 | -0.17 | -0.09 | 1.08** | 0.03 |
| \|t-ratio\| |  |  | 1.13 | | 1.06 | 0.91 | 0.63 | 2.86 | 0.84 |
| Diabetes 12m |  |  | -0.16 | | 0.24 | 0.26 | 0.15 | -0.37 | -0.04 |
| \|t-ratio\| |  |  | 1.10 | | 1.06 | 1.37 | 1.10 | 1.03 | 1.12 |
| R^2^ |  |  | 0.31 | | 0.21 | 0.29 | 0.25 | 0.32 | 0.33 |
| P-all zero^a^ |  |  | <0.001 | | <0.001 | <0.001 | <0.001 | <0.001 | <0.001 |
| P-diabetes^b^ |  |  | 0.916 | | 0.940 | 0.592 | 0.610 | 0.028 | 0.798 |
| Note: t-ratios are based on robust standard errors. *** p < 0.001, ** p < 0.01, * p < 0.05. Models are estimated as specified in eq (1). All models include a constant, year-dummies, month-dummies and individual fixed effects. Number of observations=350 325 Number of individuals=6 266 ^a^P-all zero refers to F-test of the null hypothesis that all coefficients are equal to zero. ^b^P-diabetes refers to F-test of the null hypothesis that *Diabetes* + *Diabetes12m* = 0 | | | | | | | | | |

Table S4. Changes in dietary consumption after a T2D diagnosis (Single households only)

|  | Overall diet healthiness |  | Energy share from food groups | | | |  | | Energy share from unhealthy food groups | | | |
| --- | --- | --- | --- | --- | --- | --- | --- | --- | --- | --- | --- | --- |
|  | HEI |  | Fruit & vegetables | Fish | Meat |  | | SSB^c^ | | Cakes | Candy |  |
| Diabetes | 1.26*** |  | 0.68* | 0.20 | -0.22 |  | | 0.06 | | 0.15 | -0.30 |  |
| \|t-ratio\| | 3.73 |  | 2.36 | 1.53 | 0.58 |  | | 0.28 | | 0.60 | 1.38 |  |
| Diabetes 12m | -1.72*** |  | -0.68 | -0.42* | 0.79* |  | | -0.01 | | 0.36 | 0.28 |  |
| \|t-ratio\| | 5.24 |  | 1.94 | 3.02 | 2.02 |  | | 0.05 | | 1.46 | 1.44 |  |
| R^2^ | 0.41 |  | 0.42 | 0.25 | 0.35 |  | | 0.42 | | 0.33 | 0.35 |  |
| P-all zero^a^ | <0.001 |  | <0.001 | <0.001 | <0.001 |  | | <0.001 | | <0.001 | <0.001 |  |
| P-diabetes^b^ | 0.110 |  | 0.985 | 0.025 | 0.073 |  | | 0.759 | | 0.004 | 0.946 |  |
| Note: t-ratios are based on robust standard errors. *** p < 0.001, ** p < 0.01, * p < 0.05. Models are estimated as specified in eq (1). All models include a constant, year-dummies, month-dummies and individual fixed effects. Number of observations=81 685 Number of individuals=1 443 ^a^P-all zero refers to F-test of the null hypothesis that all coefficients are equal to zero. ^b^P-diabetes refers to F-test of the null hypothesis that *Diabetes* + *Diabetes12m* = 0 ^a^ Sugar sweetened beverages. ^c^SSB = Sugar Sweetnend Beverages | | | | | | | | | | | |  |

Table S4. Cont.

|  |  |  | | Energy from nutrients | | | | | |
| --- | --- | --- | --- | --- | --- | --- | --- | --- | --- |
|  |  |  | Protein | | Unsaturated fat | Saturated fat | Added sugar | Carbohydrates | Fiber |
| Diabetes |  |  | 0.47** | | 0.52 | -0.41 | -0.01 | 0.41 | 0.11** |
| \|t-ratio\| |  |  | 2.57 | | 1.76 | 1.78 | 0.06 | 0.91 | 2.62 |
| Diabetes 12m |  |  | 0.03 | | -0.17 | 0.79 | 0.45* | -0.12 | -0.08* |
| \|t-ratio\| |  |  | 0.17 | | 0.60 | 3.55 | 2.35 | 0.28 | 2.09 |
| R^2^ |  |  | 0.33 | | 0.22 | 0.31 | 0.26 | 0.35 | 0.37 |
| P-all zero^a^ |  |  | <0.001 | | <0.001 | <0.001 | <0.001 | <0.001 | <0.001 |
| P-diabetes^b^ |  |  | 0.001 | | 0.146 | 0.060 | 0.025 | 0.467 | 0.528 |
| Note: t-ratios are based on robust standard errors. *** p < 0.001, ** p < 0.01, * p < 0.05. Models are estimated as specified in eq (1). All models include a constant, year-dummies, month-dummies and individual fixed effects. Number of observations=81 685 Number of individuals=1 443 ^a^P-all zero refers to F-test of the null hypothesis that all coefficients are equal to zero. ^b^P-diabetes refers to F-test of the null hypothesis that *Diabetes* + *Diabetes12m* = 0. | | | | | | | | | |

Table S5. Changes in dietary consumption after a T2D diagnosis, including 1 month lead variable

|  | Overall diet healthiness |  | Energy share from food groups | | |  | | Energy share from unhealthy food groups | | |  |
| --- | --- | --- | --- | --- | --- | --- | --- | --- | --- | --- | --- |
|  | HEI |  | Fruit & vegetables | Fish | Meat |  | SSB^b^ | | Cakes | Candy | |
| Diabetes | 1.33** |  | 0.87 | 0.09 | -0.72 |  | -0.24 | | -0.17 | -0.13 | |
| \|t-ratio\| | 2.619 |  | 1.72 | 0.47 | 0.85 |  | 0.82 | | 0.55 | 0.52 | |
| Pre-diabetes (1month) | -0.27 |  | 0.20 | -0.01 | -0.10 |  | 0.32 | | 0.08 | 0.07 | |
| \|t-ratio\| | 0.46 |  | 0.41 | 0.03 | 0.12 |  | 1.11 | | 0.27 | 0.28 | |
| Diabetes 12m | -1.40*** |  | -0.53** | -0.29*** | 0.31 |  | -0.06 | | 0.23* | 0.32*** | |
| \|t-ratio\| | 7.61 |  | 2.87 | 3.94 | 1.44 |  | 0.65 | | 2.32 | 3.81 | |
| R^2^ | 0.40 |  | 0.40 | 0.30 | 0.33 |  | 0.33 | | 0.30 | 0.34 | |
| P-all zero^a^ | <0.001 |  | <0.001 | <0.001 | <0.001 |  | <0.001 | | <0.001 | <0.001 | |
| Note: t-ratios are based on robust standard errors. *** p < 0.001, ** p < 0.01, * p < 0.05. Models are estimated as specified in eq (1). All models include a constant, year-dummies, month-dummies and individual fixed effects. Number of observations=359 680 Number of individuals=6 303 ^a^P-all zero refers to F-test of the null hypothesis that all coefficients are equal to zero. ^b^ Sugar sweetened beverages. | | | | | | | | | | | |

Table S5. Cont.

|  |  | | Energy share from nutrients | | | | | |
| --- | --- | --- | --- | --- | --- | --- | --- | --- |
|  |  | Protein | | Unsaturated fat | Saturated fat | Added sugar | Carbo-hydrates | Fiber |
| Diabetes |  | 0.51 | | 0.56 | -0.66 | 0.38 | 0.31 | 0.06 |
| \|t-ratio\| |  | 1.78 | | 1.34 | 1.82 | 1.61 | 0.39 | 0.08 |
| Pre-diabetes (1month) |  | -0.20 | | -0.78 | 0.10 | -0.02 | 0.43 | 0.03 |
| \|t-ratio\| |  | 0.71 | | 1.95 | 0.28 | 0.11 | 0.56 | 0.42 |
| Diabetes 12m |  | -0.24** | | 0.12 | 0.69*** | 0.07 | -0.19 | -0.07*** |
| \|t-ratio\| |  | 2.58 | | 0.81 | 5.83 | 0.72 | 0.79 | 3.53 |
| R^2^ |  | 0.31 | | 0.21 | 0.29 | 0.25 | 0.32 | 0.33 |
| P-all zero^a^ |  | <0.001 | | <0.001 | <0.001 | <0.001 | <0.001 | <0.001 |
| Note: t-ratios are based on robust standard errors. *** p < 0.001, ** p < 0.01, * p < 0.05. Models are estimated as specified in eq (1). All models include a constant, year-dummies, month-dummies and individual fixed effects. Number of observations=359 680 Number of individuals=6 303 ^a^P-all zero refers to F-test of the null hypothesis that all coefficients are equal to zero. | | | | | | | | |

Table S6. Changes in dietary consumption after a T2D diagnosis, including 2 month lead variable

|  | Overall diet healthiness |  | Energy share from food groups | | |  | | Energy share from unhealthy food groups | | | |
| --- | --- | --- | --- | --- | --- | --- | --- | --- | --- | --- | --- |
|  | HEI |  | Fruit & vegetables | Fish | Meat |  | SSB^b^ | | Cakes | Candy |  |
| Diabetes | 1.04* |  | 1.02** | 0.11 | -1.07 |  | -0.18 | | -0.03 | -0.09 |  |
| \|t-ratio\| | 2.37 |  | 2.95 | 0.76 | -1.77 |  | -0.83 | | -0.13 | -0.46 |  |
| Pre-diabetes (2month) | -0.02 |  | -0.01 | -0.03 | 0.25 |  | 0.26 | | -0.08 | -0.11 |  |
| \|t-ratio\| | -0.05 |  | -0.02 | -0.23 | 0.42 |  | 1.28 | | -0.38 | -0.63 |  |
| Diabetes 12m | -1.38*** |  | -0.54** | -0.29*** | 0.30 |  | -0.05 | | 0.23* | 0.32*** |  |
| \|t-ratio\| | 7.46 |  | -2.89 | -3.94 | 1.39 |  | -0.55 | | 2.32 | 3.74 |  |
| R^2^ | 0.40 |  | 0.40 | 0.30 | 0.33 |  | 0.33 | | 0.31 | 0.34 |  |
| P-all zero^a^ | <0.001 |  | <0.001 | <0.001 | <0.001 |  | <0.001 | | <0.001 | <0.001 |  |
| Note: t-ratios are based on robust standard errors. *** p < 0.001, ** p < 0.01, * p < 0.05. Models are estimated as specified in eq (1). All models include a constant, year-dummies, month-dummies and individual fixed effects. Number of observations=353 456 Number of individuals=6 173 ^a^P-all zero refers to F-test of the null hypothesis that all coefficients are equal to zero. ^b^ Sugar sweetened beverages. | | | | | | | | | | |  |

Table S6. Cont.

|  |  | | Energy share from nutrients | | | | | |
| --- | --- | --- | --- | --- | --- | --- | --- | --- |
|  |  | Protein | | Unsaturated fat | Saturated fat | Added sugar | Carbo-hydrates | Fiber |
| Diabetes |  | 0.16 | | 0.35 | -0.30 | 0.47** | 0.10 | 0.05 |
| \|t-ratio\| |  | 0.75 | | 1.05 | -1.13 | 2.71 | 0.17 | 0.89 |
| Pre-diabetes (2month) |  | 0.16 | | -0.60 | -0.24 | -0.13 | 0.60 | 0.04 |
| \|t-ratio\| |  | 0.78 | | -1.90 | -0.97 | -0.84 | 1.10 | 0.86 |
| Diabetes 12m |  | -0.25** | | 0.10 | 0.66*** | 0.06 | -0.14 | -0.07*** |
| \|t-ratio\| |  | -2.64 | | 0.69 | 5.50 | 0.65 | -0.58 | -3.52 |
| R^2^ |  | 0.31 | | 0.22 | 0.29 | 0.25 | 0.32 | 0.33 |
| P-all zero^a^ |  | <0.001 | | <0.001 | <0.001 | <0.001 | <0.001 | <0.001 |
| Note: t-ratios are based on robust standard errors. *** p < 0.001, ** p < 0.01, * p < 0.05. Models are estimated as specified in eq (1). All models include a constant, year-dummies, month-dummies and individual fixed effects. Number of observations=353 456 Number of individuals=6 173 ^a^P-all zero refers to F-test of the null hypothesis that all coefficients are equal to zero. | | | | | | | | |

Table S7. Changes in dietary consumption after a T2D diagnosis, including 3 month lead variable

|  | Overall diet healthiness |  | Energy share from food groups | | |  | | Energy share from unhealthy food groups | | | |
| --- | --- | --- | --- | --- | --- | --- | --- | --- | --- | --- | --- |
|  | HEI |  | Fruit & vegetables | Fish | Meat |  | SSB^b^ | | Cakes | Candy |  |
| Diabetes | 1.31*** |  | 0.94** | 0.15 | -1.04* |  | -0.16 | | -0.06 | -0.01 |  |
| \|t-ratio\| | 3.40 |  | 2.90 | 1.27 | -2.20 |  | -0.88 | | -0.36 | -0.05 |  |
| Pre-diabetes (3month) | -0.33 |  | 0.05 | -0.08 | 0.24 |  | 0.26 | | -0.03 | -0.21 |  |
| \|t-ratio\| | -0.91 |  | 0.19 | 0.80 | 0.52 |  | 1.57 | | -0.17 | -1.34 |  |
| Diabetes 12m | -1.37*** |  | -0.56** | -0.29*** | 0.33 |  | -0.06 | | 0.21* | 0.32*** |  |
| \|t-ratio\| | -7.33 |  | -2.98 | -3.87 | 1.50 |  | -0.64 | | 2.10 | 3.79 |  |
| R^2^ | 0.40 |  | 0.41 | 0.30 | 0.33 |  | 0.33 | | 0.31 | 0.34 |  |
| P-all zero^a^ | <0.001 |  | <0.001 | <0.001 | <0.001 |  | <0.001 | | <0.001 | <0.001 |  |
| Note: t-ratios are based on robust standard errors. *** p < 0.001, ** p < 0.01, * p < 0.05. Models are estimated as specified in eq (1). All models include a constant, year-dummies, month-dummies and individual fixed effects. Number of observations=347 252 Number of individuals=5 968 ^a^P-all zero refers to F-test of the null hypothesis that all coefficients are equal to zero. ^b^ Sugar sweetened beverages. | | | | | | | | | | |  |

Table S7. Cont.

|  |  | | Energy share from nutrients | | | | | |
| --- | --- | --- | --- | --- | --- | --- | --- | --- |
|  |  | Protein | | Unsaturated fat | Saturated fat | Added sugar | Carbo-hydrates | Fiber |
| Diabetes |  | 0.11 | | 0.21 | -0.33 | 0.28 | -0.13 | 0.07 |
| \|t-ratio\| |  | 0.58 | | 0.73 | -1.42 | 1.55 | -0.26 | 1.50 |
| Pre-diabetes (3month) |  | 0.22 | | -0.45 | -0.21 | 0.07 | 0.76 | 0.02 |
| \|t-ratio\| |  | 1.23 | | -1.71 | 0.96 | 0.42 | 1.66 | 0.53 |
| Diabetes 12m |  | -0.24* | | 0.12 | 0.65*** | 0.02 | -0.17 | -0.08*** |
| \|t-ratio\| |  | -2.48 | | 0.84 | 5.43 | 0.24 | -0.70 | -3.60 |
| R^2^ |  | 0.31 | | 0.21 | 0.29 | 0.25 | 0.32 | 0.33 |
| P-all zero^a^ |  | <0.001 | | <0.001 | <0.001 | <0.001 | <0.001 | <0.001 |
| Note: t-ratios are based on robust standard errors. *** p < 0.001, ** p < 0.01, * p < 0.05. Models are estimated as specified in eq (1). All models include a constant, year-dummies, month-dummies and individual fixed effects. Number of observations=347 252 Number of individuals=5 968 ^a^P-all zero refers to F-test of the null hypothesis that all coefficients are equal to zero | | | | | | | | |

Table S8. Changes in dietary consumption after a T2D diagnosis, including 6 month lead variable

|  | Overall diet healthiness |  | Energy share from food groups | | |  | | Energy share from unhealthy food groups | | | | |
| --- | --- | --- | --- | --- | --- | --- | --- | --- | --- | --- | --- | --- |
|  | HEI |  | Fruit & vegetables | Fish | Meat |  | SSB^b^ | | Cakes | Candy |  |  |
| Diabetes | 1.25 |  | 1.02 | 0.13 | -0.77 |  | -0.02 | | 0.03 | -0.08 |  |  |
| \|t-ratio\| | 4.19 |  | 3.82 | 1.30 | -2.15 |  | -0.16 | | 0.21 | -0.59 |  |  |
| Pre-diabetes (6month) | -0.37 |  | -0.11 | -0.07 | -0.01 |  | 0.19 | | -0.16 | -0.16 |  |  |
| \|t-ratio\| | -1.39 |  | -0.52 | -0.98 | -0.04 |  | 1.45 | | -1.28 | -1.32 |  |  |
| Diabetes 12m | -1.37 |  | -0.71 | -0.30 | 0.33 |  | -0.11 | | 0.21 | 0.31 |  |  |
| \|t-ratio\| | -7.22 |  | -3.75 | -3.87 | 1.50 |  | -1.11 | | 2.14 | 3.68 |  |  |
| R^2^ | 0.40 |  | 0.41 | 0.30 | 0.33 |  | 0.34 | | 0.31 | 0.34 |  |  |
| P-all zero^a^ | <0.001 |  | <0.001 | <0.001 | <0.001 |  | <0.001 | | <0.001 | <0.001 |  |  |
| Note: t-ratios are based on robust standard errors. *** p < 0.001, ** p < 0.01, * p < 0.05. Models are estimated as specified in eq (1). All models include a constant, year-dummies, month-dummies and individual fixed effects. Number of observations=347 252 Number of individuals=5 968 ^a^P-all zero refers to F-test of the null hypothesis that all coefficients are equal to zero. ^b^ Sugar sweetened beverages. | | | | | | | | | | | |  |

Table S8. Cont.

|  |  | | Energy share from nutrients | | | | | |  |
| --- | --- | --- | --- | --- | --- | --- | --- | --- | --- |
|  |  | Protein | | Unsat Fat | Sat fat | Carb | Fiber | Add sugar |  |
| Diabetes |  | 0.17 | | -0.06 | -0.50 | 0.15 | 0.07 | 0.36 |  |
| \|t-ratio\| |  | 1.18 | | -0.25 | -2.74 | 0.40 | 2.03 | 2.63 |  |
| Pre-diabetes (6month) |  | 0.16 | | -0.15 | 0.02 | 0.27 | 0.01 | -0.03 |  |
| \|t-ratio\| |  | 1.26 | | -0.77 | 0.11 | 0.85 | 0.26 | -0.28 |  |
| Diabetes 12m |  | -0.24 | | 0.13 | 0.67 | -0.23 | -0.08 | -0.04 |  |
| \|t-ratio\| |  | -2.44 | | 0.89 | 5.54 | -0.94 | -3.57 | -0.41 |  |
| R^2^ |  | 0.31 | | 0.22 | 0.29 | 0.32 | 0.33 | 0.25 |  |
| P-all zero^a^ |  | <0.001 | | <0.001 | <0.001 | <0.001 | <0.001 | <0.001 |  |
| Note: t-ratios are based on robust standard errors. *** p < 0.001, ** p < 0.01, * p < 0.05. Models are estimated as specified in eq (1). All models include a constant, year-dummies, month-dummies and individual fixed effects. Number of observations=329988 Number of individuals=5 610 ^a^P-all zero refers to F-test of the null hypothesis that all coefficients are equal to zero | | | | | | | | | |

Table S9a. Changes in dietary consumption after a T2D diagnosis, including interactions between diagnosed individuals in pre-diagnosis time periods and time variables (year and month)

|  | HEI |  | Fruit & veg. | Fish | Meat |  | SSB | Cakes | Candy |
| --- | --- | --- | --- | --- | --- | --- | --- | --- | --- |
| Diabetes | 0.01^***^ |  | 0.91^*^ | 0.10 | -0.73 |  | -0.26 | -0.18 | -0.15 |
| \|t-ratio\| | 2.24 |  | 1.81 | 0.50 | -0.86 |  | -0.88 | -0.59 | -0.58 |
| Diabetes 12m | -0.01^***^ |  | -0.48^***^ | -0.27^***^ | 0.24 |  | -0.07 | 0.23^***^ | 0.32^***^ |
| \|t-ratio\| | -7.27 |  | -2.62 | -3.73 | 1.11 |  | -0.69 | 2.40 | 3.80 |
| Year |  |  |  |  |  |  |  |  |  |
| 2007 | 0.03^*^ |  | 0.00 | -0.11 | -0.02 |  | 0.01 | 0.01 | 0.01^*^ |
| \|t-ratio\| | 1.91 |  | 0.07 | -1.12 | -0.69 |  | 1.60 | 0.66 | 1.86 |
| 2008 | 0.04^**^ |  | 0.00 | -0.11 | -0.02 |  | 0.00 | 0.00 | 0.01^***^ |
| \|t-ratio\| | 2.09 |  | 0.25 | -1.13 | -0.65 |  | 1.04 | 0.49 | 4.66 |
| 2009 | 0.03^**^ |  | 0.00 | -0.11 | -0.03 |  | 0.00 | 0.00 | 0.01^***^ |
| \|t-ratio\| | 1.98 |  | 0.28 | -1.13 | -0.72 |  | 0.75 | 0.42 | 5.07 |
| 2010 | 0.03^*^ |  | 0.00 | -0.11 | -0.03 |  | 0.00 | 0.00 | 0.02^***^ |
| \|t-ratio\| | 1.94 |  | 0.27 | -1.13 | -0.77 |  | 0.85 | 0.58 | 5.59 |
| 2011 | 0.03^*^ |  | 0.00 | -0.11 | -0.03 |  | 0.00 | 0.01 | 0.02^***^ |
| \|t-ratio\| | 1.85 |  | 0.14 | -1.14 | -0.87 |  | 1.04 | 0.82 | 5.86 |
| 2012 | 0.03^**^ |  | 0.00 | -0.11 | -0.03 |  | 0.00 | 0.01 | 0.02^***^ |
| \|t-ratio\| | 2.02 |  | 0.19 | -1.13 | -0.87 |  | 0.74 | 0.77 | 5.52 |
| 2013 | 0.03^*^ |  | 0.00 | -0.11 | -0.03 |  | 0.00 | 0.01 | 0.02^***^ |
| \|t-ratio\| | 1.65 |  | 0.06 | -1.13 | -0.83 |  | 1.06 | 0.87 | 5.46 |
| 2014 | 0.03^*^ |  | 0.00 | -0.11 | -0.03 |  | 0.01 | 0.01 | 0.02^***^ |
| \|t-ratio\| | 1.71 |  | 0.10 | -1.14 | -0.94 |  | 1.57 | 0.91 | 5.21 |
| 2015 | 0.03^*^ |  | 0.00 | -0.11 | -0.04 |  | 0.01 | 0.01 | 0.01^***^ |
| \|t-ratio\| | 1.81 |  | 0.11 | -1.13 | -1.01 |  | 1.56 | 0.91 | 4.73 |
| 2016 | 0.03^*^ |  | 0.00 | -0.11 | -0.04 |  | 0.01^**^ | 0.01 | 0.02^***^ |
| \|t-ratio\| | 1.70 |  | -0.18 | -1.13 | -1.13 |  | 2.09 | 1.09 | 5.35 |
| 2017 | 0.03^*^ |  | -0.01 | -0.11 | -0.05 |  | 0.00 | 0.00 | 0.01^***^ |
| \|t-ratio\| | 1.82 |  | -0.47 | -1.15 | -1.48 |  | -0.80 | 0.63 | 4.53 |

Table S9a. Cont.

|  | HEI |  | Fruit & veg. | Fish | Meat |  | SSB | Cakes | Candy |
| --- | --- | --- | --- | --- | --- | --- | --- | --- | --- |
| Month |  |  |  |  |  |  |  |  |  |
| 2 | -0.01^***^ |  | 0.00^***^ | 0.00^***^ | 0.00^***^ |  | 0.00^***^ | 0.00^***^ | 0.00^***^ |
| \|t-ratio\| | -25.75 |  | -10.41 | 2.21 | 4.77 |  | 5.97 | 5.08 | 17.27 |
| 3 | -0.01^***^ |  | -0.01^***^ | 0.01^***^ | 0.00^***^ |  | 0.00^***^ | 0.00^***^ | 0.01^***^ |
| \|t-ratio\| | -26.27 |  | -16.84 | 23.74 | 5.14 |  | 3.83 | 8.55 | 32.90 |
| 4 | -0.02^***^ |  | -0.01^***^ | 0.00^***^ | 0.00^***^ |  | 0.00^***^ | 0.00^***^ | 0.01^***^ |
| \|t-ratio\| | -32.94 |  | -20.01 | 15.94 | 6.37 |  | 11.26 | 18.12 | 27.99 |
| 5 | -0.02^***^ |  | -0.01^***^ | 0.00^***^ | 0.00^***^ |  | 0.00^***^ | 0.01^***^ | 0.00^***^ |
| \|t-ratio\| | -37.49 |  | -21.33 | 3.83 | 7.72 |  | 8.57 | 29.61 | 12.18 |
| 6 | -0.02^***^ |  | -0.01^***^ | 0.00^***^ | 0.01^***^ |  | 0.00^***^ | 0.01^***^ | 0.00^***^ |
| \|t-ratio\| | -42.89 |  | -22.10 | 7.05 | 10.72 |  | 13.03 | 34.95 | 6.22 |
| 7 | -0.03^***^ |  | -0.01^***^ | 0.00^***^ | 0.00^***^ |  | 0.01^***^ | 0.01^***^ | 0.00^***^ |
| \|t-ratio\| | -46.72 |  | -24.59 | -0.71 | 7.53 |  | 17.48 | 32.98 | 2.29 |
| 8 | -0.03^***^ |  | -0.01^***^ | 0.00^***^ | 0.00^***^ |  | 0.00^***^ | 0.01^***^ | 0.00^***^ |
| \|t-ratio\| | -43.09 |  | -29.39 | 4.00 | 6.05 |  | 11.02 | 18.50 | 0.74 |
| 9 | -0.03^***^ |  | -0.02^***^ | 0.00^***^ | 0.00^***^ |  | 0.00^***^ | 0.00^***^ | 0.00^***^ |
| \|t-ratio\| | -46.11 |  | -36.10 | 10.45 | 5.73 |  | 4.87 | 15.61 | 13.74 |
| 10 | -0.03^***^ |  | -0.02^***^ | 0.00^***^ | 0.01^***^ |  | 0.00^***^ | 0.00^***^ | 0.01^***^ |
| \|t-ratio\| | -46.66 |  | -34.76 | -1.28 | 8.64 |  | 1.91 | 16.87 | 25.84 |
| 11 | -0.03^***^ |  | -0.02^***^ | 0.00^***^ | 0.00^***^ |  | 0.00^***^ | 0.01^***^ | 0.01^***^ |
| \|t-ratio\| | -54.48 |  | -34.93 | 2.20 | 4.53 |  | -4.30 | 22.45 | 42.78 |
| 12 | -0.04^***^ |  | -0.02^***^ | 0.01^***^ | 0.01^***^ |  | 0.00^***^ | 0.01^***^ | 0.02^***^ |
| \|t-ratio\| | -69.63 |  | -47.74 | 34.70 | 8.27 |  | 4.28 | 20.30 | 61.04 |

Table S9a. Cont.

|  | HEI |  | Fruit & veg. | Fish | Meat |  | SSB | Cakes | Candy |
| --- | --- | --- | --- | --- | --- | --- | --- | --- | --- |
| Parallel Year |  |  |  |  |  |  |  |  |  |
| 2007 | 0.01 |  | 0.00 | 0.00 | 0.00 |  | 0.00 | 0.00 | 0.00 |
| \|t-ratio\| | 1.47 |  | -0.17 | -0.01 | -0.47 |  | -1.54 | -0.18 | 0.43 |
| 2008 | 0.01 |  | 0.00 | 0.00 | 0.00 |  | 0.00 | 0.00 | 0.00 |
| \|t-ratio\| | 0.74 |  | -0.16 | -0.67 | 0.21 |  | -0.66 | -0.56 | 0.25 |
| 2009 | 0.00 |  | 0.00 | 0.00 | 0.00 |  | 0.00 | 0.00 | 0.00 |
| \|t-ratio\| | 0.59 |  | -0.08 | -0.91 | -0.14 |  | -0.34 | -0.29 | 1.02 |
| 2010 | 0.00 |  | 0.00 | 0.00 | 0.00 |  | 0.00 | -0.01 | 0.00 |
| \|t-ratio\| | 0.53 |  | -0.78 | -1.16 | -0.13 |  | -0.20 | -1.63 | 0.23 |
| 2011 | 0.01 |  | 0.00 | 0.00 | 0.00 |  | 0.00 | 0.00 | 0.00 |
| \|t-ratio\| | 0.87 |  | -0.37 | -1.20 | -0.44 |  | -0.54 | -1.10 | 1.16 |
| 2012 | 0.00 |  | -0.01 | 0.00 | 0.00 |  | 0.00 | 0.00 | 0.00 |
| \|t-ratio\| | 0.43 |  | -1.31 | -1.12 | -0.36 |  | -0.72 | -1.42 | 1.35 |
| 2013 | -0.01 |  | -0.01 | 0.00 | 0.01 |  | 0.00 | 0.00 | 0.00 |
| \|t-ratio\| | -0.78 |  | -1.19 | -1.40 | 0.74 |  | -0.40 | -1.27 | 1.10 |
| 2014 | 0.00 |  | -0.01 | 0.00 | 0.00 |  | 0.00 | -0.01 | 0.00 |
| \|t-ratio\| | -0.21 |  | -1.43 | -1.10 | 0.20 |  | -0.36 | -1.58 | 0.49 |
| 2015 | 0.00 |  | -0.01^*^ | -0.01^**^ | 0.01 |  | 0.00 | 0.00 | 0.00 |
| \|t-ratio\| | -0.31 |  | -1.93 | -2.32 | 0.66 |  | -0.38 | 0.33 | 0.33 |
| 2016 | 0.00 |  | -0.01 | 0.00^**^ | 0.02 |  | 0.00 | 0.00 | 0.00 |
| \|t-ratio\| | -0.20 |  | -0.97 | -2.04 | 1.57 |  | -0.76 | 0.25 | -0.24 |
| 2017 | -0.02^*^ |  | -0.01 | -0.01^***^ | 0.02^*^ |  | 0.00 | 0.00 | 0.01 |
| \|t-ratio\| | -1.92 |  | -1.33 | -2.96 | 1.82 |  | 0.51 | -0.51 | 1.25 |

Table S9a. Cont.

|  | HEI |  | Fruit & veg. | Fish | Meat |  | SSB | Cakes | Candy |
| --- | --- | --- | --- | --- | --- | --- | --- | --- | --- |
| Parallel Month |  |  |  |  |  |  |  |  |  |
| 1 | 0.00 |  | 0.00 | 0.00^**^ | 0.00 |  | 0.00 | 0.00 | 0.00 |
| \|t-ratio\| | 0.40 |  | 0.00 | 2.31 | -0.39 |  | -1.00 | 0.79 | -1.27 |
| 2 | 0.00 |  | 0.00 | 0.00^*^ | 0.00 |  | 0.00 | 0.00 | 0.00 |
| \|t-ratio\| | -1.26 |  | 0.05 | 1.79 | 0.02 |  | -0.62 | 1.22 | -1.00 |
| 3 | 0.00 |  | 0.00 | 0.00 | 0.00 |  | 0.00^**^ | 0.00^*^ | 0.00 |
| \|t-ratio\| | -0.45 |  | 0.02 | 0.83 | -0.36 |  | -2.08 | 1.78 | -0.14 |
| 4 | 0.00 |  | 0.00 | 0.00^***^ | 0.00 |  | 0.00 | 0.00 | 0.00^***^ |
| \|t-ratio\| | 0.41 |  | -0.78 | 2.38 | 0.23 |  | -0.51 | 1.31 | -1.97 |
| 5 | 0.00 |  | 0.00 | 0.00^***^ | 0.00 |  | 0.00 | 0.00 | 0.00 |
| \|t-ratio\| | -0.56 |  | -0.13 | 2.40 | -0.22 |  | -0.09 | 0.91 | -0.14 |
| 6 | 0.00 |  | 0.00 | 0.00^*^ | 0.00 |  | 0.00 | 0.00 | 0.00 |
| \|t-ratio\| | -0.24 |  | 1.44 | 1.77 | 0.58 |  | 0.72 | -0.13 | -1.00 |
| 7 | 0.00 |  | 0.00 | 0.00^***^ | 0.00 |  | 0.00 | 0.00 | 0.00 |
| \|t-ratio\| | -0.73 |  | 0.99 | 2.46 | 0.69 |  | -1.52 | 0.82 | -1.26 |
| 8 | 0.00 |  | 0.00 | 0.00^***^ | 0.00 |  | 0.00 | 0.00 | 0.00 |
| \|t-ratio\| | -0.20 |  | 0.58 | 2.59 | 1.00 |  | -0.06 | 1.11 | 0.03 |
| 9 | 0.00 |  | 0.00 | 0.00 | 0.00 |  | 0.00 | 0.00 | 0.00 |
| \|t-ratio\| | 0.33 |  | 0.98 | 1.19 | 0.40 |  | -0.73 | 0.32 | -1.20 |
| 10 | 0.00 |  | 0.01^**^ | 0.00^***^ | 0.00 |  | 0.00 | 0.00^***^ | 0.00 |
| \|t-ratio\| | -0.27 |  | 2.09 | 2.62 | 0.28 |  | -1.16 | 2.37 | -0.66 |
| 11 | 0.00 |  | 0.00 | 0.00 | 0.00 |  | 0.00 | 0.00 | 0.00 |
| \|t-ratio\| | -0.23 |  | 0.60 | 0.78 | -0.04 |  | -1.08 | 1.54 | -0.24 |
| Constant | 0.76^***^ |  | 7.51^***^ | 11.89 | 14.14^***^ |  | 1.34^***^ | 1.42^*^ | 0.51^*^ |
| \|t-ratio\| | 44.30 |  | 4.51 | 1.27 | 4.04 |  | 3.55 | 1.79 | 1.77 |
| R^2^ | 0.40 |  | 0.40 | 0.29 | 0.33 |  | 0.33 | 0.30 | 0.34 |
| Parallel Year & Month = 0 | 0.040 |  | 0.057 | 0.001 | 0.034 |  | 0.112 | 0.003 | 0.453 |

Table S9b. Changes in dietary consumption after a T2D diagnosis, including interactions between diagnosed individuals in pre-diagnosis time periods and time variables (year and month)

|  | Protein | Unsaturated fat | Saturated fat | Added sugar | Carbo-hydrates | Fiber |
| --- | --- | --- | --- | --- | --- | --- |
| Diabetes | 0.48^*^ | 0.52 | -0.71^*^ | 0.38 | 0.33 | 0.06 |
| \|t-ratio\| | 1.68 | 1.26 | -1.94 | 1.61 | 0.41 | 0.77 |
| Diabetes 12m | -0.26^***^ | 0.13 | 0.66^***^ | 0.08 | -0.21 | -0.07^***^ |
| \|t-ratio\| | -2.71 | 0.88 | 5.57 | 0.87 | -0.85 | -3.25 |
| Year |  |  |  |  |  |  |
| 2007 | 0.00 | -0.03 | 0.01 | 0.01^*^ | 0.06 | 0.01 |
| \|t-ratio\| | -0.04 | -0.87 | 0.22 | 1.70 | 1.22 | 1.53 |
| 2008 | 0.00 | -0.03 | 0.00 | 0.01 | 0.07 | 0.01 |
| \|t-ratio\| | -0.09 | -0.90 | 0.12 | 1.92 | 1.31 | 1.59 |
| 2009 | 0.00 | -0.03 | 0.00 | 0.01^**^ | 0.07 | 0.01 |
| \|t-ratio\| | -0.03 | -0.90 | 0.10 | 1.96 | 1.34 | 1.60 |
| 2010 | 0.00 | -0.03 | 0.00 | 0.02^**^ | 0.07 | 0.01 |
| \|t-ratio\| | -0.03 | -0.86 | 0.14 | 2.06 | 1.30 | 1.50 |
| 2011 | 0.00 | -0.03 | 0.00 | 0.02^**^ | 0.07 | 0.01 |
| \|t-ratio\| | -0.13 | -0.85 | 0.13 | 2.16 | 1.35 | 1.48 |
| 2012 | 0.00 | -0.03 | 0.00 | 0.02^**^ | 0.07 | 0.01 |
| \|t-ratio\| | -0.05 | -0.84 | 0.16 | 2.14 | 1.29 | 1.54 |
| 2013 | 0.00 | -0.03 | 0.01 | 0.02^**^ | 0.06 | 0.01 |
| \|t-ratio\| | -0.06 | -0.75 | 0.29 | 2.16 | 1.22 | 1.49 |
| 2014 | -0.01 | -0.03 | 0.01 | 0.02^***^ | 0.06 | 0.01 |
| \|t-ratio\| | -0.26 | -0.72 | 0.28 | 2.36 | 1.23 | 1.49 |
| 2015 | -0.04 | 0.01 | 0.01 | 0.01^***^ | 0.00 | 0.01 |
| \|t-ratio\| | -1.01 | 1.56 | 0.91 | 4.73 | -0.54 | 3.54 |
| 2016 | -0.04 | 0.01^**^ | 0.01 | 0.02^***^ | 0.00 | 0.01^***^ |
| \|t-ratio\| | -1.13 | 2.09 | 1.09 | 5.35 | -0.86 | 3.95 |
| 2017 | -0.05 | 0.00 | 0.00 | 0.01 | 0.00^***^ | 0.00^***^ |
| \|t-ratio\| | -1.48 | -0.80 | 0.63 | 4.53 | -2.43 | 3.28 |

Table S9b. Cont.

|  | Protein | Unsaturated fat | Saturated fat | Added sugar | Carbo-hydrates | Fiber |
| --- | --- | --- | --- | --- | --- | --- |
| Month |  |  |  |  |  |  |
| 2 | -0.01^***^ | 0.00^***^ | 0.01^***^ | 0.00^***^ | 0.00^***^ | 0.00^***^ |
| \|t-ratio\| | -20.29 | 7.23 | 17.92 | 9.40 | -6.18 | -24.40 |
| 3 | -0.01^***^ | 0.01^***^ | 0.01^***^ | 0.00^***^ | -0.02^***^ | 0.00^***^ |
| \|t-ratio\| | -25.92 | 18.45 | 30.70 | 8.18 | -22.64 | -29.36 |
| 4 | -0.01^***^ | 0.01^***^ | 0.01^***^ | 0.00^***^ | -0.01^***^ | 0.00^***^ |
| \|t-ratio\| | -26.11 | 13.94 | 27.53 | 14.04 | -17.75 | -33.34 |
| 5 | -0.01^***^ | 0.00^***^ | 0.01^***^ | 0.00^***^ | -0.01^***^ | 0.00^***^ |
| \|t-ratio\| | -29.00 | 6.26 | 16.33 | 15.42 | -12.55 | -41.59 |
| 6 | -0.01^***^ | 0.00^***^ | 0.00^***^ | 0.00^***^ | -0.01^***^ | 0.00^***^ |
| \|t-ratio\| | -34.57 | 3.32 | 14.02 | 14.83 | -14.43 | -50.61 |
| 7 | -0.01 | 0.00 | 0.00^***^ | 0.00^***^ | -0.01^***^ | 0.00^***^ |
| \|t-ratio\| | -28. ^***^63 | -0.30 | 8.31 | 8.24 | -6.72 | -51.05 |
| 8 | -0.01^***^ | 0.00^***^ | 0.00^***^ | 0.00^***^ | -0.01^***^ | 0.00^***^ |
| \|t-ratio\| | -27.13 | 4.66 | 11.07 | 1.47 | -9.47 | -42.43 |
| 9 | -0.01^***^ | 0.00^***^ | 0.01^***^ | 0.00^***^ | -0.01^***^ | 0.00^***^ |
| \|t-ratio\| | -35.13 | 7.50 | 20.80 | 3.54 | -12.35 | -47.92 |
| 10 | -0.01^***^ | 0.00^***^ | 0.01^***^ | 0.00^***^ | -0.01^***^ | 0.00^***^ |
| \|t-ratio\| | -30.23 | 3.46 | 18.93 | 10.29 | -7.88 | -43.47 |
| 11 | -0.01^***^ | 0.01^***^ | 0.01^***^ | 0.00^***^ | -0.01^***^ | 0.00^***^ |
| \|t-ratio\| | -36.32 | 14.64 | 30.88 | 9.46 | -17.30 | -52.69 |
| 12 | -0.02^***^ | 0.01^***^ | 0.02^***^ | 0.00 | -0.02^***^ | 0.00^***^ |
| \|t-ratio\| | -63.48 | 20.72 | 54.85 | 0.26 | -33.28 | -77.07 |

Table S9b. Cont.

|  | Protein | Unsaturated fat | Saturated fat | Added sugar | Carbo-hydrates | Fiber |
| --- | --- | --- | --- | --- | --- | --- |
| Parallel Year |  |  |  |  |  |  |
| 2007 | 0.01^**^ | 0.01^*^ | 0.00 | 0.00 | -0.01 | 0.00 |
| \|t-ratio\| | 2.19 | 1.91 | -0.92 | -0.42 | -0.60 | 0.55 |
| 2008 | 0.01^*^ | 0.01 | 0.00 | 0.00 | 0.00 | 0.00 |
| \|t-ratio\| | 1.75 | 1.46 | -0.69 | -0.24 | -0.42 | 0.53 |
| 2009 | 0.01^*^ | 0.01 | 0.00 | 0.00 | -0.01 | 0.00 |
| \|t-ratio\| | 1.83 | 1.44 | -0.60 | -0.03 | -0.67 | 0.43 |
| 2010 | 0.00 | 0.01 | 0.00 | 0.00 | 0.00 | 0.00 |
| \|t-ratio\| | 1.12 | 1.60 | -0.69 | -0.56 | -0.41 | -0.01 |
| 2011 | 0.00 | 0.01 | 0.00 | 0.00 | 0.00 | 0.00 |
| \|t-ratio\| | 1.26 | 1.24 | -0.21 | 0.69 | 0.22 | 0.24 |
| 2012 | 0.00 | 0.01 | 0.00 | 0.00 | 0.00 | 0.00 |
| \|t-ratio\| | 1.19 | 1.47 | -0.43 | -0.71 | 0.10 | -0.27 |
| 2013 | 0.00 | 0.01^*^ | 0.00 | 0.00 | 0.00 | 0.00 |
| \|t-ratio\| | 1.22 | 1.65 | 0.39 | 0.25 | -0.17 | -0.37 |
| 2014 | 0.01^**^ | 0.01^*^ | 0.00 | -0.01^**^ | 0.00 | 0.00 |
| \|t-ratio\| | 1.96 | 1.82 | -0.06 | -2.07 | -0.19 | -0.42 |
| 2015 | 0.01^***^ | 0.00 | 0.00 | 0.00 | 0.00 | 0.00 |
| \|t-ratio\| | 3.01 | 0.61 | -0.61 | -1.20 | 0.23 | -0.49 |
| 2016 | 0.01^***^ | 0.01 | 0.00 | 0.00 | 0.00 | 0.00 |
| \|t-ratio\| | 2.51 | 1.01 | 0.25 | -1.08 | -0.43 | -0.03 |
| 2017 | 0.02^***^ | 0.02^**^ | 0.01 | 0.00 | 0.01 | 0.00 |
| \|t-ratio\| | 3.17 | 2.07 | 0.74 | 0.46 | 0.54 | -0.40 |

Table S9b. Cont.

|  | Protein | Unsaturated fat | Saturated fat | Added sugar | Carbo-hydrates | Fiber |
| --- | --- | --- | --- | --- | --- | --- |
| Parallel Month |  |  |  |  |  |  |
| 1 | -0.01^***^ | 0.00^*^ | 0.00 | 0.00 | 0.00 | 0.00 |
| \|t-ratio\| | -2.93 | -1.80 | -1.37 | 0.44 | 0.93 | -1.12 |
| 2 | -0.01^***^ | 0.00 | 0.00 | 0.00 | 0.00 | 0.00^**^ |
| \|t-ratio\| | -4.32 | 0.88 | 1.06 | -0.34 | -1.01 | -2.28 |
| 3 | 0.00^***^ | 0.00 | 0.00 | 0.00 | 0.00 | 0.00^**^ |
| \|t-ratio\| | -2.60 | -0.29 | -0.82 | 0.54 | -0.29 | -2.13 |
| 4 | 0.00^***^ | 0.00 | 0.00 | 0.00 | 0.00 | 0.00^*^ |
| \|t-ratio\| | -2.60 | -0.61 | -0.12 | -0.10 | -0.24 | -1.75 |
| 5 | 0.00^***^ | 0.00 | 0.00 | 0.00 | 0.00 | 0.00 |
| \|t-ratio\| | -2.07 | 0.70 | 1.06 | 1.02 | -0.88 | -1.47 |
| 6 | 0.00^*^ | 0.00 | 0.00 | 0.00^*^ | 0.00 | 0.00 |
| \|t-ratio\| | -1.67 | 0.97 | 0.41 | 1.88 | -0.30 | -0.99 |
| 7 | 0.00 | 0.00 | 0.01^***^ | 0.00 | -0.01^**^ | 0.00^**^ |
| \|t-ratio\| | -0.46 | 1.04 | 2.21 | 1.06 | -2.25 | -2.28 |
| 8 | 0.00 | 0.00 | 0.00 | 0.00^**^ | 0.00 | 0.00 |
| \|t-ratio\| | -1.49 | 0.45 | 1.15 | 2.28 | -0.84 | -1.07 |
| 9 | 0.00^***^ | 0.00 | 0.00 | 0.00 | 0.00 | 0.00 |
| \|t-ratio\| | -2.06 | -0.06 | 0.16 | 0.59 | -0.09 | 0.40 |
| 10 | 0.00 | 0.00 | 0.00 | 0.00^*^ | 0.00 | 0.00 |
| \|t-ratio\| | -1.33 | 0.04 | 0.97 | 1.80 | -0.30 | -0.35 |
| 11 | -0.01^***^ | 0.00 | 0.00 | 0.00 | 0.00 | 0.00 |
| \|t-ratio\| | -3.27 | 0.32 | -0.30 | 0.03 | 0.47 | 0.02 |
| Constant | 15.71^***^ | 21.36^***^ | 14.29^***^ | 2.92^***^ | 37.37^***^ | 1.89^***^ |
| \|t-ratio\| | 7.69 | 5.70 | 5.91 | 4.01 | 7.21 | 5.35 |
| R^2^ | 0.31 | 0.21 | 0.29 | 0.25 | 0.32 | 0.33 |
| Parallel Year & Month=0 | <0.001 | 0.312 | 0.117 | 0.007 | 0.473 | 0.100 |

Table S10. Changes in dietary consumption after a T2D diagnosis. Diagnosed Individuals only

|  | Overall diet healthiness |  | Energy share from food groups | | |  | | Energy share from unhealthy food groups | | |  |
| --- | --- | --- | --- | --- | --- | --- | --- | --- | --- | --- | --- |
|  | HEI |  | Fruit & vegetables | Fish | Meat |  | SSB^c^ | | Cakes | Candy | |
| Diabetes | 1.27*** |  | 1.09*** | 0.12 | -0.65*** |  | 0.02 | | -0.13 | -0.36* | |
| \|t-ratio\| | 5.64 |  | 5.52 | 1.18 | 3.55 |  | 0.59 | | -1.09 | -2.36 | |
| Diabetes 12m | -1.16*** |  | -0.53** | -0.25*** | 0.35 |  | -0.11 | | 0.20* | 0.18*** | |
| \|t-ratio\| | 7.62 |  | 2.87 | 3.97 | 1.35 |  | -0.62 | | 2.31 | 3.87 | |
| R^2^ | 0.40 |  | 0.40 | 0.29 | 0.33 |  | 0.33 | | 0.30 | 0.34 | |
| P-all zero^a^ | <0.001 |  | <0.001 | <0.001 | <0.001 |  | <0.001 | | <0.001 | <0.001 | |
| P-diabetes^b^ | 0.027 |  | <0.001 | <0.001 | 0.006 |  | 0.964 | | 0.119 | 0.132 | |
| Note: t-ratios are based on robust standard errors. *** p < 0.001, ** p < 0.01, * p < 0.05. Models are estimated as specified in eq (1). All models include a constant, year-dummies, month-dummies and individual fixed effects. Number of observations=366 036 Number of individuals=6 430 ^a^P-all zero refers to F-test of the null hypothesis that all coefficients are equal to zero. ^b^P-diabetes refers to F-test of the null hypothesis that *Diabetes* + *Diabetes12m* = 0. ^c^ Sugar sweetened beverages. | | | | | | | | | | | |

Table S10. Cont.

|  |  | | Energy share from nutrients | | | | | |
| --- | --- | --- | --- | --- | --- | --- | --- | --- |
|  |  | Protein | | Unsaturated fat | Saturated fat | Added sugar | Carbo-hydrates | Fiber |
| Diabetes |  | 0.44*** | | -0.11 | -0.61*** | 0.29*** | 0.14** | 0.12*** |
| \|t-ratio\| |  | 3.22 | | 1.47 | 4.88 | 3.71 | 3.11 | 4.20 |
| Diabetes 12m |  | -0.10** | | 0.24 | 0.64*** | -0.01 | -0.79 | -0.05*** |
| \|t-ratio\| |  | 2.63 | | 0.87 | 5.74 | 0.73 | 0.76 | 3.47 |
| R^2^ |  | 0.31 | | 0.21 | 0.29 | 0.25 | 0.32 | 0.33 |
| P-all zero^a^ |  | <0.001 | | <0.001 | <0.001 | <0.001 | <0.001 | <0.001 |
| P-diabetes^b^ |  | 0.448 | | 0.416 | 0.335 | <0.001 | 0.003 | 0.316 |
| Note: t-ratios are based on robust standard errors. *** p < 0.001, ** p < 0.01, * p < 0.05. Models are estimated as specified in eq (1). All models include a constant, year-dummies, month-dummies and individual fixed effects. Number of observations=366 036 Number of individuals=6 430 ^a^P-all zero refers to F-test of the null hypothesis that all coefficients are equal to zero. ^b^P-diabetes refers to F-test of the null hypothesis that *Diabetes* + *Diabetes12m* = 0 | | | | | | | | |

Table S11. Change in diet six months after diagnosis compared to six months prior to diagnosis (age and income as continuous, baseline as categorical)

|  | Overall healthiness |  | Energy percentage from food groups | | |  | Energy percentage from unhealthy food groups | | |
| --- | --- | --- | --- | --- | --- | --- | --- | --- | --- |
|  | HEI |  | Fruit & veg. | Fish | Meat |  | SSB | Cakes | Candy |
| Female | -0.005 |  | 0.012 | 0.002 | 0.003 |  | -0.001 | -0.003 | 0.000 |
| (\|t- ratio\|) | -0.690 |  | 1.380 | 1.030 | 0.390 |  | -0.180 | -0.780 | -0.120 |
| Single household | 0.019^***^ |  | -0.010 | 0.001 | -0.008 |  | 0.001 | 0.000 | 0.003 |
| (\|t- ratio\|) | 2.510 |  | -1.010 | 0.420 | -1.000 |  | 0.300 | -0.040 | 0.740 |
| Age | 0.000 |  | 0.000 | 0.000 | 0.000 |  | 0.000 | 0.000 | 0.000 |
| (\|t- ratio\|) | -1.010 |  | 0.260 | 1.060 | 0.920 |  | -0.510 | 0.240 | -1.650 |
| Ln(income) | 0.006 |  | 0.000 | 0.004^*^ | -0.003 |  | -0.003 | 0.001 | -0.002 |
| (\|t- ratio\|) | 0.940 |  | -0.040 | 1.810 | -0.400 |  | -0.830 | 0.170 | -0.750 |
| Baseline_q25 | 0.022^***^ |  | 0.004 | 0.001 | 0.038^***^ |  | 0.008^*^ | 0.008 | 0.003 |
| (\|t- ratio\|) | 2.710 |  | 0.380 | 0.280 | 4.570 |  | 1.830 | 1.950^*^ | 0.680 |
| Baseline_q75 | -0.030^***^ |  | 0.000 | -0.003 | -0.045^***^ | ^******^ | -0.014^***^ | -0.023 | -0.018^***^ |
| (\|t- ratio\|) | -3.850 |  | -0.020 | -1.230 | -5.380 |  | -3.090 | -4.950^***^ | -4.480 |
| Constant | -0.044 |  | 0.004 | -0.049^*^ | 0.015 |  | 0.040 | -0.004 | 0.041 |
| (\|t- ratio\|) | -0.570 |  | 0.040 | -1.890 | 0.180 |  | 0.890 | -0.100 | 1.050 |
| Observations | 261 |  | 261 | 261 | 261 | 261 | 261 | 261 | 261 |
| R2 | 0.14 |  | 0.01 | 0.03 | 0.23 |  | 0.07 | 0.14 | 0.10 |
| F-statistic | 6.62 |  | 0.43 | 1.25 | 12.93 |  | 3.23 | 6.82 | 4.76 |
| Note: Income is included in logarithmic form. T-ratios are based on robust standard errors. F-statistic of the null hypothesis that all coefficients are equal to zero. *** p < 0.001, ** p < 0.01, * p < 0.05. N=261 | | | | | | | | | |

Table S11 continued

|  |  | Energy percentage from nutrients | | | | | |
| --- | --- | --- | --- | --- | --- | --- | --- |
|  |  | Protein | Unsat Fat | Sat fat | Carb | Fiber | Add sugar |
| Female |  | 0.004 | -0.003 | 0.001 | 0.007 | 0.001 | 0.002 |
| (\|t- ratio\|) |  | 1.080 | -0.590 | 0.160 | 0.890 | 0.920 | 0.540 |
| Single household |  | -0.001 | -0.001 | -0.001 | -0.002 | 0.000 | 0.001 |
| (\|t- ratio\|) |  | -0.200 | -0.270 | -0.210 | -0.210 | 0.470 | 0.280 |
| Age |  | 0.000 | 0.000 | 0.000^***^ | 0.000 | 0.000 | 0.000 |
| (\|t- ratio\|) |  | -0.340 | 0.050 | 2.220 | -1.240 | -0.810 | -1.140 |
| Ln(income) |  | 0.004 | 0.000 | 0.003 | -0.009 | 0.000 | -0.004 |
| (\|t- ratio\|) |  | 1.320 | 0.060 | 0.920 | -1.300 | -0.170 | -1.110 |
| Baseline_q25 |  | 0.005 | 0.016^***^ | 0.007 | 0.018^**^ | 0.001^*^ | 0.003 |
| (\|t- ratio\|) |  | 1.160 | 3.180 | 1.580 | 1.990 | 1.660 | 0.640 |
| Baseline_q75 |  | -0.012^***^ | -0.021^***^ | -0.022^***^ | -0.028^***^ | -0.004^***^ | -0.011^***^ |
| (\|t- ratio\|) |  | -3.070 | -4.020 | -4.730 | -3.070 | -5.340 | -2.450 |
| Constant |  | -0.045 | -0.004 | -0.063 | 0.138 | 0.004 | 0.061 |
| (\|t- ratio\|) |  | -1.140 | -0.080 | -1.390 | 1.550 | 0.440 | 1.360 |
| Observations |  | 261 | 261 | 261 | 261 | 261 | 261 |
| R2 |  | 0.07 | 0.14 | 0.13 | 0.10 | 0.15 | 0.05 |
| F-statistic |  | 3.06 | 6.86 | 6.35 | 4.76 | 7.52 | 2.09 |
| Note: Income is included in logarithmic form. T-ratios are based on robust standard errors. ^a^F-statistic of the null hypothesis that all coefficients are equal to zero. *** p < 0.001, ** p < 0.01, * p < 0.05. N=261 | | | | | | | |

Table S12. Change in diet six months after diagnosis compared to six months prior to diagnosis

|  | Overall healthiness |  | Energy percentage from food groups | | |  | Energy percentages from unhealthy food groups | | |
| --- | --- | --- | --- | --- | --- | --- | --- | --- | --- |
|  | HEI |  | Fruit & veg. | Fish | Meat |  | SSB^b^ | Cakes | Candy |
| Female | -0.002 |  | 0.013 | 0.002 | 0.004 |  | -0.005 | 0.001 | 0.002 |
| (\|t- ratio\|) | -0.330 |  | 1.500 | 0.800 | 0.580 |  | -1.220 | 0.520 | 0.680 |
| Single household | 0.018* |  | -0.009 | 0.001 | -0.011 |  | 0.005 | 0.004 | 0.004 |
| (\|t- ratio\|) | 2.560 |  | 0.990 | 0.500 | 1.500 |  | 1.230 | 1.320 | 1.350 |
| Age | 0.002 |  | 0.002 | 0.000 | 0.001 |  | -0.002* | -0.001 | -0.001* |
| (\|t- ratio\|) | 1.300 |  | 1.270 | 0.630 | 0.920 |  | 1.980 | 1.110 | 2.460 |
| Income | -0.020 |  | 0.100 | 0.025 | -0.081 |  | 0.102 | -0.017 | -0.050 |
| (\|t- ratio\|) | 0.150 |  | 0.580 | 0.540 | 0.570 |  | 1.350 | 0.320 | 0.890 |
| Age^2^ | 0.000 |  | 0.000 | 0.000 | 0.000 |  | 0.000 | 0.000 | 0.000* |
| (\|t- ratio\|) | 1.400 |  | 1.260 | 0.430 | 0.760 |  | 1.780 | 1.190 | 2.320 |
| Income^2^ | 0.001 |  | -0.004 | -0.001 | 0.003 |  | -0.004 | 0.001 | 0.002 |
| (\|t- ratio\|) | 0.190 |  | -0.610 | -0.460 | 0.550 |  | -1.360 | 0.390 | 0.880 |
| Baseline | -1.415* |  | -0.354* | -0.467*** | -0.691*** |  | -0.260*** | -0.221** | -0.032 |
| (\|t- ratio\|) | 2.120 |  | 2.050 | 3.950 | 5.920 |  | 2.550 | 3.260 | 0.450 |
| Baseline^2^ | 0.758 |  | 1.800** | 4.291*** | 0.712** |  | -0.589 | -2.398*** | -1.948*** |
| (\|t- ratio\|) | 1.700 |  | 3.090 | 4.040 | 2.670 |  | 1.050 | 8.030 | 5.790 |
| Constant | 0.688 |  | -0.613 | -0.182 | 0.534 |  | -0.553 | 0.104 | 0.346 |
| (\|t- ratio\|) | 0.820 |  | 0.600 | 0.660 | 0.630 |  | 1.220 | 0.330 | 1.030 |
| R2 | 0.19 |  | 0.06 | 0.08 | 0.28 |  | 0.18 | 0.60 | 0.40 |
| F-statistic^a^ | 7.23 |  | 2.11 | 2.87 | 12.33 |  | 6.78 | 46.62 | 21.32 |
| Note: Income is included in logarithmic form. T-ratios are based on robust standard errors. ^a^F-statistic of the null hypothesis that all coefficients are equal to zero. ^b^ SSB = Sugar Sweetened Beverages*** p < 0.001, ** p < 0.01, * p < 0.05. N=261 | | | | | | | | | |

Table S12. Change in diet six months after diagnosis compared to six months prior to diagnosis

|  |  | Energy percentages from nutrients | | | | | |
| --- | --- | --- | --- | --- | --- | --- | --- |
|  |  | Protein | Unsat.Fat | Sat. fat | Carb | Fiber | Added sugar |
| Female |  | 0.004 | -0.001 | 0.002 | 0.004 | 0.001 | 0.001 |
| (\|t- ratio\|) |  | 1.260 | 0.330 | 0.590 | 0.540 | 0.930 | 0.320 |
| Single household |  | -0.001 | -0.002 | 0.000 | 0.001 | 0.000 | 0.002 |
| (\|t- ratio\|) |  | 0.200 | 0.340 | 0.030 | 0.100 | 0.510 | 0.400 |
| Age |  | 0.001 | 0.001 | 0.000 | -0.003 | 0.000 | 0.000 |
| (\|t- ratio\|) |  | 1.610 | 0.770 | 0.540 | 1.770 | 1.220 | 0.330 |
| Income |  | -0.052 | -0.009 | -0.052 | 0.170 | -0.015 | 0.027 |
| (\|t- ratio\|) |  | 0.770 | 0.110 | 0.680 | 1.090 | 0.990 | 0.350 |
| Age^2^ |  | 0.000 | 0.000 | 0.000 | 0.000 | 0.000 | 0.000 |
| (\|t- ratio\|) |  | 1.710 | 0.880 | 0.300 | 1.510 | 1.280 | 0.600 |
| Income^2^ |  | 0.002 | 0.000 | 0.002 | -0.007 | 0.001 | -0.001 |
| (\|t- ratio\|) |  | 0.800 | 0.090 | 0.690 | 1.140 | 0.970 | 0.390 |
| Baseline |  | -0.450 | -0.112 | 0.481* | -0.026 | -0.483** | -0.163 |
| (\|t- ratio\|) |  | 1.280 | 0.480 | 2.340 | 0.090 | 3.120 | 0.950 |
| Baseline^2^ |  | 0.684 | -0.774 | -2.403*** | -0.231 | 3.820 | -0.857 |
| (\|t- ratio\|) |  | 0.600 | 1.340 | 4.160 | 0.640 | 1.370 | 0.820 |
| Constant |  | 0.323 | 0.100 | 0.270 | -0.831 | 0.092 | -0.131 |
| (\|t- ratio\|) |  | 0.780 | 0.190 | 0.590 | 0.880 | 1.050 | 0.280 |
| R2 |  | 0.09 | 0.23 | 0.23 | 0.12 | 0.15 | 0.08 |
| F-statistic^a^ |  | 3.14 | 9.52 | 9.57 | 4.20 | 5.72 | 2.63 |
| Note: Income is included in logarithmic form. t-ratios are based on robust standard errors. ^a^F-statistic of the null hypothesis that all coefficients are equal to zero. *** p < 0.001, ** p < 0.01, * p < 0.05. N=261 | | | | | | | |
